# Supplementary material for: Protective factors and sources of support in the workplace as experienced by UK foundation and junior doctors: a qualitative study
Source: BMJ Open. 2021 Jun 23;11(6):e045588. doi: 10.1136/bmjopen-2020-045588 (PMC8231035; doi:10.1136/bmjopen-2020-045588)
Supplement: Author's manuscript [file bmjopen-2020-045588.draft_revisions.pdf]

BMJ Open is committed to open peer review. As part of this commitment we make the peer review history of every article we publish publicly available.

When an article is published we post the peer reviewers' comments and the authors' responses online. We also post the versions of the paper that were used during peer review. These are the versions that the peer review comments apply to.

The versions of the paper that follow are the versions that were submitted during the peer review process. They are not the versions of record or the final published versions. They should not be cited or distributed as the published version of this manuscript.

BMJ Open is an open access journal and the full, final, typeset and author-corrected version of record of the manuscript is available on our site with no access controls, subscription charges or pay-per-view fees (http://bmjopen.bmj.com).

If you have any questions on BMJ Open's open peer review process please email info.bmjopen@bmj.com

BMJ Open

Protective factors and sources of support in the workplace as experienced by UK foundation and junior doctors: a qualitative study

Journal:	*BMJ Open*
Manuscript ID	bmjopen-2020-045588
Article Type:	Original research
Date Submitted by the Author:	07-Oct-2020
Complete List of Authors:	Riley, Ruth; University of Birmingham College of Medical and Dental Sciences<br>Kokab, Farina ; University of Birmingham,<br>Buszewicz, Marta; University College London, Research Department of Primary Care and Population Health<br>Gopfert, Anya; Oxford University Hospitals NHS Trust<br>Van Hove, Maria; London School of Hygiene & Tropical Medicine<br>Taylor, Anna<br>Teoh, Kevin; Birkbeck University of London, Department of Organizational Psychology<br>Martin, James; University of Birmingham, Institute of Applied Health Research<br>Appleby, Louis; University of Manchester, Psychiatry & Behavioral Sciences<br>Chew-Graham, Carolyn; University of Keele
Keywords:	QUALITATIVE RESEARCH, OCCUPATIONAL & INDUSTRIAL MEDICINE, MENTAL HEALTH, MEDICAL EDUCATION & TRAINING, Suicide & self-harm < PSYCHIATRY

SCHOLARONE™
Manuscripts

I, the Submitting Author has the right to grant and does grant on behalf of all authors of the Work (as defined in the below author licence), an exclusive licence and/or a non-exclusive licence for contributions from authors who are: i) UK Crown employees; ii) where BMJ has agreed a CC-BY licence shall apply, and/or iii) in accordance with the terms applicable for US Federal Government officers or employees acting as part of their official duties; on a worldwide, perpetual, irrevocable, royalty-free basis to BMJ Publishing Group Ltd ("BMJ") its licensees and where the relevant Journal is co-owned by BMJ to the co-owners of the Journal, to publish the Work in this journal and any other BMJ products and to exploit all rights, as set out in our licence.

The Submitting Author accepts and understands that any supply made under these terms is made by BMJ to the Submitting Author unless you are acting as an employee on behalf of your employer or a postgraduate student of an affiliated institution which is paying any applicable article publishing charge ("APC") for Open Access articles. Where the Submitting Author wishes to make the Work available on an Open Access basis (and intends to pay the relevant APC), the terms of reuse of such Open Access shall be governed by a Creative Commons licence – details of these licences and which Creative Commons licence will apply to this Work are set out in our licence referred to above.

Other than as permitted in any relevant BMJ Author's Self Archiving Policies, I confirm this Work has not been accepted for publication elsewhere, is not being considered for publication elsewhere and does not duplicate material already published. I confirm all authors consent to publication of this Work and authorise the granting of this licence.

For peer review only

Protective factors and sources of support in the workplace as experienced by UK foundation and junior doctors: a qualitative study

Ruth Riley < Corresponding author>

Institute of Applied Health Research

University of Birmingham
Birmingham

United Kingdom

r.riley@bham.ac.uk

Tel: + 44 (0) 121 414 8549

Farina Kokab,

Department of Social Work and Social Policy

School of Social Policy

University of Birmingham

Birmingham

United Kingdom

Marta Buszewicz,

Research Department of Primary Care and Population Health

University College London

London

United Kingdom

Anya Gopfert

For peer review only

Oxford University Hospitals Trust

Oxford

United Kingdom

Maria van Hove

London School of Hygiene and Tropical Medicine

London

United Kingdom

Anna Kathryn Taylor

Leeds Institute of Health Sciences

Faculty of Medicine and Health

University of Leeds

Leeds

United Kingdom

Kevin Rui-Han Teoh,

Department of Organizational Psychology
Birkbeck, University of London

London

United Kingdom

James Martin

Institute of Applied Health Research

For peer review only

University of Birmingham
Birmingham

United Kingdom

Louis Appleby

Division of Psychology and Mental Health

School of Medicine

University of Manchester

Manchester

United Kingdom

Carolyn A. Chew-Graham

School of Medicine

Keele University

Keele

United Kingdom

For peer review only

Abstract

Objectives. This paper reports findings identifying foundation and junior doctors' experiences of occupational and psychological protective factors and sources of effective support in the workplace.

Design. Interpretative, qualitative study involving in-depth interviews with 21 junior doctor participants. The interviews were audio-recorded, transcribed, anonymised and imported into NVivo11 to facilitate data management. Data were analysed using a thematic analysis employing the constant comparative method.

Setting. NHS in UK.

Participants. A purposive sample of junior doctor participants who self-identified as having stress, distress, anxiety, depression and suicidal thoughts or having attempted to kill themselves.

Results. Analysis identified three main themes, with corresponding subthemes relating to protective work factors and facilitators of support: 1. Peer support – help with managing workloads and emotional support. 2. Supportive leadership strategies, including feeling valued and accepted, trust and communication, supportive learning environments and challenging stigma and normalising vulnerability 3. Access to professional support – counselling, CBT and medication through General Practitioners, specialist support for doctors, and private therapy.

Conclusions.

Findings show that supportive leadership, effective management practices, peer support, and access to appropriate professional support can help mitigate the negative impact of working conditions and cultures experienced by junior doctors. Feeling connected, supported and valued by colleagues and consultants acts as an important buffer against emotional distress despite working under challenging working conditions.

Keywords: Qualitative, Hospital Staff, Occupational Stress, Mental Health, Suicide, Psychological Support

Word count: 2762

For peer review only

Strengths and limitations of this study

- Few qualitative studies have explored psychological distress in junior doctors. We employed in-depth interviews to explore sources of distress, sources of support and protective factors, interviewing 21 junior doctors from across the UK. This study contributes to research examining and identifying perceived protective factors and effective sources of support experienced by junior doctors.

- The analysis was conducted by a multidisciplinary team comprising three junior doctors, two academic clinicians, an occupational health psychologist and three social scientists which afforded checks and balances to the validity of the analytic process, interpretation of data and transferability of the research findings.

- Participants were self-selecting, which may be perceived as a limitation; however, the sampling of participants who expressed an interest in the study, ensured that the sample was varied in terms of ethnicity, number of years in training, speciality, and geographical location.

- The sample included five male participants and 16 female participants, which may be a limitation in this study; we received more expressions of interest from female (n=37) participants compared to male (n=22), with a greater number of female participants taking up interviews as reflected in the interview sample. The interest in this study and increased willingness to come forward amongst female participants may reflect evidence indicating that female doctors are more likely to report and experience distress, with increased rates of suicides also being reported in female doctors. The interest may also reflect gender differences in help-seeking behaviour, evidenced in the general population.

INTRODUCTION

There are currently 115,376 doctors working in the NHS, almost half of whom (56,404) are 'junior doctors', including, doctors in training or at pre-Consultant grade, and foundation doctors [1]. Previous research indicates that doctors are vulnerable to chronic stress, anxiety, depression and burnout [2, 3]. High rates of suicide have frequently been reported but with considerable heterogeneity over time and between countries [4]. Despite mixed findings about suicide rates internationally, there are concerns over suicidal ideation and behaviour in doctors, particularly amongst female doctors [5, 6].

For peer review only

The Practitioner Health Programme in London, an England-wide specialist mental health, drug and alcohol service for doctors and dentists, reports that two-thirds of those presenting are women, with higher attendance rates among young female doctors [7]. It is uncertain whether this is representative of the general population of doctors or reflects gender differences in help-seeking behaviour.

Most previous research into distress and suicide in junior doctors reports quantitative, retrospective studies, with the focus on the individual (particularly emphasising the need for 'resilience'), [8, 9] and overlooking the wider systemic, organisational and cultural sources of distress among this population of health professionals. This approach is likely to detract from considering solutions/interventions which target organisational culture and systemic factors [10]. To our knowledge, no previous study has qualitatively examined how junior doctors view their working conditions and work cultures, and the factors (e.g. debriefs following critical incidents, policies, available support, management practices) which may protect them from psychological distress or offer them support.

Junior doctors face specific pressures related to their professional stage and development. In a nationwide survey of junior doctors in Ireland, stress, anxiety and depression were highest among junior doctors as compared to consultants [11]. A study exploring junior doctors' reasons for leaving medicine in the UK cited lack of support, mentorship or formal training, loneliness and bullying [12]. A lack of support, mental health stigma, and toxic work cultures, characterised by a bullying and a blame and shame culture were identified as a key source of distress among junior doctors, in a separate paper linked to this study [Riley et al. 2020 – under review BMJ Open]. Feeling undervalued, unsupported or having reduced autonomy were key factors affecting low morale among junior doctors [13]; this evidence highlights the impact of working conditions and work culture on junior doctors and the potential value of providing effective support and supervision.

Evidence has found that junior doctors valued the emotional support derived from attending debriefing sessions following adverse events, although its effectiveness in reducing burnout was equivocal [14]. Workplace interventions such as Schwartz rounds, which provide permission and a safe space for healthcare staff to talk about the range of feelings and challenges which arise during their work while also fostering connectedness to others, were valued by staff [15]. Although numerous workplace health theories [16, 17] identify support as a core antecedent to positive mental health and as a potential buffer against challenging work environments, how this might manifest amongst junior doctors is not clear. Identifying such patterns or contextual factors is crucial in developing targeted improvements or interventions that can reduce the risk of mental ill health and psychological distress from the outset.

This qualitative study is part of a wider mixed-methods study examining the psychological, cultural and occupational contexts associated with reduced

For peer review only

psychological wellbeing in junior doctors, and protective factors which may mitigate against this. This paper reports the qualitative findings focussed on the protective factors and sources of effective support in the workplace as experienced by junior doctors.

METHODS

Study design and setting

The study methodology was underpinned by a constructivist grounded theory approach [18], using qualitative methods, with semi-structured interviews to explore junior doctors' perspectives and experiences of stress and distress. The study setting was NHS UK.

Sampling and Recruitment

We used a range of recruitment methods including advertisements on social media (for example, the junior doctors' Facebook group, Twitter and the mental health research charity websites). In addition, information about the study was circulated through the Practitioner Health Programme, a specialised mental health and drug and alcohol service dedicated to doctors and dentists [19].

Potential participants were asked to express their interest by contacting members of the study team who then provided further information about the study.

The research team employed purposive sampling from the responding junior doctors, to ensure maximum variation taking account of the following characteristics: gender, age, ethnicity, sexuality, geographical location, different grades/duration of General Medical Council registration, medical speciality, disclosure of a mental health diagnosis, individuals who reported self-harm behaviour, had thoughts of suicide or had attempted to kill themselves.

The eligibility criteria used are listed in Box 1.

Junior doctors interested in taking part were sent a reply slip and consent form electronically and asked to sign the consent form prior to the interview, before returning it to the study researcher. Face-to-face, telephone or 'virtual platform' interviews were arranged at a time convenient to each participant. They were given the opportunity to raise questions prior to being interviewed and during the interview.

At the end of each interview, the researcher informed the participant about the next steps, how their data would be used, and checked on their wellbeing prior to leaving the interview setting. A risk protocol was used to ensure appropriate support was

For peer review only

provided to participants (and the researcher) in the event of the disclosure of significant distress or suicidal ideation.

Data collection

A topic guide (see box 2) was developed by the research team to generate discussion in the semi-structured interviews. The topic guide was informed by the existing literature, input from junior doctors on the study team, and patient and public involvement (PPI) input (see below), and modified iteratively as data collection and analysis progressed. The topic guide aimed to capture participants' views, experiences, feelings and beliefs about working conditions and cultures which were perceived to be stressful or distressing, and factors which were perceived/felt to be protective.

One-to-one interviews were conducted face-to-face, by telephone or using a digital virtual platform and digitally recorded with consent. The in-depth interviews were conducted by two researchers (FK, RR), female social and behavioural scientists with qualitative methods expertise. The recorded interviews were transcribed verbatim and checked for accuracy of transcription by the study researcher before analysis. All transcripts were anonymised before discussion within the wider research team. Reflexive notes were recorded throughout the process. Recruitment and data collection was continued until data saturation was judged to have been achieved [20].

Data analysis

Analysis began with data collection, and was iterative, employing the constant comparative method [21, 22] until theoretical data saturation was achieved, such that no new analytic categories emerged [20].

The research team included three junior doctors (AG, AKT, MvH), two academic general practitioners (CCG, MB), one occupational health psychologist (KT) and two social scientists (RR and FK). FK coded the data set; RR coded a sub-sample and contributed to the organisation of themes. The multidisciplinary team provided commentary on transcripts to generate and refine codes and thematic categories and provide researcher triangulation, thereby increasing the credibility of the research findings [23]. Data were managed using NVivo 11. The study is reported in line with the consolidated criteria for reporting qualitative research (COREQ) [24].

Patient and Public Involvement

There were three junior doctors on the research team, all of whom consulted with colleagues about the initial research idea. Five junior doctors provided feedback on the initial funding application, and four of these gave feedback on the protocol, topic guide and participant facing documents. We are working on

For peer review only

a dissemination strategy with junior doctors outside the research team. Due to the time constraints of junior doctors, PPIE members were consulted via email and telephone.

FINDINGS

Twenty-one interviews were conducted, lasting between 43 and 103 min (mean= 65 min), between November 2019 and May 2020. The demographic and professional characteristics of participants are included in Table 1. Analysis of the interview transcripts and field notes identified three main themes, with corresponding subthemes relating to protective work factors and facilitators of support: 1. Peer support, including help with managing workloads and emotional support. 2. Supportive leadership strategies, including feeling valued and accepted, trust and communication, supportive learning environments and challenging stigma and normalising vulnerability 3. Access to professional support, including Cognitive Behavioural Therapy (CBT), counselling and medication through General Practitioner and specialist support for doctors, and attending private counselling/therapy.

PEER SUPPORT

Many participants emphasised the importance of working in protective and supportive work cultures, characterised by good interpersonal relationships, a strong team morale where there was a shared responsibility for workloads, and where staff felt supported with the clinical components of their work and the emotional impact of their job. Crucially, participants highlighted the interdependence between collegial support, supportive work cultures and their emotional wellbeing and ability to cope with the demands of their job.

a) Help with managing workloads

Participants identified the benefits of having mutually supportive interpersonal connections at work where there was a willingness to share responsibilities, offer practical support, helping with workloads or specific tasks:

"*I've made good relationships… [name of speciality] was actually having a very, very nice group of colleagues on the whole and supporting each other. Like offering help if we're struggling, if someone's struggling with a task then I would offer help and vice versa*." (JD12, male)

The following participant highlighted that nurses offered support by undertaking tasks which free up time for junior doctors to focus on clinical work: Spending time with supportive staff could alleviate some of the burden and stress that junior doctors may be experiencing, especially in new and unfamiliar roles.

"My relationships with my colleagues, both nurses and doctors, are incredible. I couldn't say a bad word about any of them. They have been so wonderfully supportive. The nursing staff have carried me in every job I've been on. They've

For peer review only - http://bmjopen.bmj.com/site/about/guidelines.xhtml

carried me because they're the constant and they're there all the time." (JD17, female)

b) Emotional support

Participants iterated the importance of peer support and feeling emotionally supported within the team, for example, in response to critical incidents, or being bullied by a colleague::

"*A couple of weeks ago my friend was having a terrible time with a registrar who was being hideous to her and so, you know we kind of helped support her through that and empathise with her and you know I think that's really helpful.*" (JD20, female)

The following participant referred to the '*in it together*' team spirit and camaraderie which acted as an emotional buffer when working under challenging working conditions:

"*We were all in it together and actually, the working culture is really, really good … even in the rubbish conditions that we're working in, it's the people that have made it worthwhile and they've helped me cope with things, so if I'm having a bad day or I'm a bit down, actually it's the camaraderie of working with midwives, other doctors, nurses, pharmacists, porters that have made it quite a happy thing to do really*." (JD09. female)

Many participants had contrasting experiences on different rotations; the following participant described her experience of being in a supportive work culture in the context of a critical incident, which she compared with the 'blame culture' experienced on a previous rotation:

"*In the unit I'm in at the moment, it's a lot more about learning rather than blaming. I think you feel safer then to express opinions or to discuss cases. You want to find out who was involved in order to be able to support them and that's the feeling that you get from the senior midwives and the consultants you work with… The unit that I was in before was really challenging in terms of feeling like you wouldn't be supported, even if you'd done nothing wrong."* (JD13, female)

Another participant reported feeling '*emotionally healthy'* which she attributed to the supportive work culture. She contrasted this with a previous rotation where the cultural norm was to disengage with challenging emotions, leaving staff with unprocessed trauma:

"I feel really emotionally healthy now. Cause I'm in such a supportive culture. But I look now particularly at [name of specialty] culture as a lot of people who, with a lot of unprocessed trauma. Huge amounts of unprocessed trauma. Amazing really that whole profession can be so in denial of the emotional impact of their job." (JD23, female)

For peer review only

The value of working in a supportive work culture was further highlighted by the following participant who recollected that having greater support from colleagues in her previous post would have reduced her sense of anxiety:

"I think if I'd have had that [informal support from colleagues] support in first year of someone who could listen, maybe I wouldn't have been so anxious about everything." (JD09, female)

The following participant suggested that, despite the potential stigma associated with mental illness, peer-support was often the first line of support for colleagues:

"I do think peer support is very important and I think that's probably the first line for a lot of people, like even with some stigma and embarrassment about mental health, I think most people would usually turn to a colleague first." (JD16, female)

SUPPORTIVE LEADERSHIP STRATEGIES

a) Feeling valued and accepted

This reoccurring theme refers to supportive and proactive leadership and management approaches to providing support to the team and promoting positive mental health. Participants relayed the importance of feeling valued, included and accepted as part of the team, by the consultants. This included receiving recognition and praise for their efforts and contributions, particularly when working in challenging, highly pressurised environments and working long shifts:

'… it [first job] was over 12 hours every single day, you know I think I started to cry because just at least someone was acknowledging the fact that we were doing 12, 13 hour days every day … it was extremely hard but…there was a very good group of consultants who just acknowledged that it was hard, there was not much they could do about it, they had to work hard as well but they were very, very, very supportive of that… they [consultants] just knew what your name was, they knew your role, you felt valued there." (JD01, male)

"…*it makes such a difference and then also like being empowered to be of good patient care, so you know not being shouted at if you've taken half an hour to see a patient and actually you know being recognised, 'Oh you did a really thorough job actually, well done, good for you' and kind of you know just actually some recognition of what you're doing. Yeah, that's what is good, I guess.*" (JD20, female)

This participant emphasised the importance of feeling valued by consultants or patients and its relationship to job satisfaction and confidence:

"…when you receive some good feedback, you can really get a bit of a buzz and a bit of a high from that, either from a patient or from a colleague when they said, you know,

For peer review only

'you've really made a difference today' or 'you've really helped me today' or 'you've made me feel better, thank you'. Whenever you get something like that, which doesn't happen all the time by any means, but when you get a bit of feedback, it makes you realise why you're doing it and I guess all those feelings of anxiety and the feeling of not being good enough, which is a big thing for me. So, if somebody's appreciative, I think that can brighten your day really and make you feel like you're worth something." (JD09, female)

b) Trust and communication

A few participants recalled the value of consultants who were proactive in their support of staff, openly inviting them to discuss concerns, ask questions or to enquire about their work and wellbeing. Establishing these open channels of communication and being approachable made it easier for participants to seek support or advice when needed:

"*Whereas, the two consultants that I go to for that sort of support always made it clear that they were open to talking about anything that was bothering me. They continued to keep that relationship open, even when I wasn't working with them anymore. It was just the odd touch base email like, 'How are things going? What are you up to? Let's have a coffee one day'. It was that kind of thing. You're made to feel a bit less like you're onerous or that you're bothering them when you ask them about something.*" (JD13, female)

Such was the importance of supportive relationship with consultants, a few participants had maintained those connections after moving to a new post:

"*I've got a couple of people that I go back to when I need support ….and I know will speak to them both but I will always go back to those few people that I trust and I know.*" (JD19, female)

c) Supportive learning environments

Participants reflected on work cultures where there was a shared commitment to team learning. Such an ethos enabled people to practice without fear of reprisal, to reflect on their work, and to view errors as opportunities for learning and improvement:

"*My ST1 [*Specialist Training year one*] year was extremely enjoyable, because again it was a very supportive place to work and they encouraged you to ask questions and didn't criticise and there was a real sort of no blame culture*" (JD01, male)

Some participants suggested that cohesiveness within teams could be enabled through multidisciplinary training opportunities. These events facilitated a shared understanding of different roles within the team and awareness of the challenges and concerns faced by different professional groups:

For peer review only

"Where I am at the moment is really good. The current unit I'm in has a lot of multi-professional training that isn't necessarily clinical. We have multi-professional leadership days where the senior junior doctors at ST5 level plus and the senior midwives all get together to do the training together. I think that really helps because we understood their role and their concerns when they're coordinating a unit and they understand that we can't really do everything" (JD13, female)

d) Challenging stigma and normalising vulnerability

A number of participants emphasised the role of consultants and supervisors in de-stigmatising mental ill health by sharing or disclosing their vulnerability or experiences of mental ill health to their staff; participants report that this created a more open and authentic work culture where staff also felt safe to disclose their vulnerability:

"We were working a Sunday together and I said, 'I haven't seen you in a while.' She said, 'No, I have been off for about four months.' I said, 'Are you just coming back?' She said, 'Yeah, I was off because I had a mental breakdown. One day, I just realised that I was really, really unwell and I just had to go home and I didn't return for a number of months until I got myself sorted out.' My point was just her talking about that, I think, does wonders because she's a woman who has been there forever. She's a senior consultant. Just telling other people, 'Yeah, I've gone through the struggles. I need medication for this, that and the other. It's okay. I'm still here functioning and it's fine'. I think people need to be more open about that," (JD03, male)

"I think what really needs to change is that I think that more senior doctors need to be open about their struggles and their issues in training because I think there's a perception that you need to be invulnerable among more junior trainees in particular, which I think as people gradually have breakdowns, they're robbed of that illusion. Yes, yes, I think that kind of cultural change needs to kind of happen." (JD07, female)

ACCESS TO PROFESSIONAL SUPPORT

Few participants reported seeking formalised help from within the workplace (e.g. occupational health) but some study participants had sought professional help from counsellors, therapists, GPs and specialist services for doctors, outside the workplace. The following participant was prescribed antidepressants by her GP to manage her distress:

"…I was in tears and nervous …I kept on saying, 'I don't feel like this is going to ever change. I don't feel confident,' and then I started antidepressants and incredibly, within about two weeks, I was sleeping. I felt better and I felt calm. I realised just how sick I had been." (JD08, female)

For peer review only

Accessing external support was not without barriers. The following participant highlights the significant delay in accessing a counselling referral through their GP:

"My GP referred me for local support and it took them a year to contact me…I mean, it's just the NHS, that's how it is…and even then, it would be one o'clock, two o'clock, it would be nigh on impossible to actually access," (JD19, female)

Having access to services tailored to the needs of junior doctors was important for participants:

"*Because you know that their [*specialist service*] whole patient group are doctors and they've got such expertise of talking to doctors and professionals with the same issues, there is none of that having to make an excuse for yourself. We're so often held up in society as a really responsible, clever and respected group that you can be vulnerable in the PHP. That was really, really validating*." (JD08, female)

The following participant found out about the Practitioner Health Programme (PHP) by chance (a family member found a PHP leaflet in a waiting room); she was concerned about being referred to a psychiatrist in her local trust because she was planning on undertaking a rotation there in the future:

"*There is the Practitioner Health Programme… I've been seeing a GP, that lives down the road from me, who's absolutely amazing…I feel like that's a really good setup…I didn't feel comfortable being treated in the mental health trust here and also, I want to look at working in that mental health trust. Being able to move my care completely away from that is very important. I didn't know that was there at the beginning. That's when I ran into some of my problems and when I wasn't seeking the help I needed because I didn't know that I could have anonymity*." (JD22, female)

Due to concerns about the potential loss of confidentiality, the following participant self-referred to a private clinic to seek the formal support they needed:

"*I went to a private mental health clinic and I referred myself, I phoned up and said well the Consultant probably would take a self-referral especially if you're a Doctor, so I wrote my own referral letter and I went privately to the Psychiatrist across the road from the hospital and he got me some treatment which was mainly in the form of counselling … And that changed everything, you know that turned everything around*." (JD15, female)

A few participants felt it would be beneficial if junior doctors had access to monthly therapy or supervision sessions, either group work or one-to-one, a safe space to share and process the emotional impact of the job and seek support:

"If, as junior doctors, you saw a therapist once a month... if we made it universal (which is very easy to say) that for one hour a month, everyone had to see someone to talk to, I think that would be a good way of moving forward. If there was something that was really serious, it's a safe space and we can talk about it. If it needs to be escalated or if any sort of treatment needs to be started in whichever way that might be, go for it." (JD03, male)

"…*sometimes lots of stuff coming up and yet there's absolutely nothing. For me I feel like, every doctor should have some form of emotional supervision.*" (JD23, female)

DISCUSSION

The findings highlight the value of peer support and work cultures engendered by camaraderie and connectedness with colleagues. The emotional support offered by colleagues often enabled junior doctors to manage the challenges of the job and was felt to be potentially protective against mental ill health. Participants felt more resilient and able to manage the challenges of their work when their managers and leaders valued staff, offering clinical and emotional support and creating positive learning environments.

Our findings also highlight the role of consultants and other senior staff in de-stigmatising mental ill health by acknowledging and sharing their own vulnerability and creating a safe and authentic work culture which enabled staff to seek support. Promoting emotion cultures which facilitate disclosure by de-stigmatising mental ill health and allowing for vulnerability, empowers staff to seek support and could reduce the sense of isolation and shame often experienced by junior doctors [25].

These findings highlight the positive impact of harnessing existing workforce capital (i.e. the skills and training of staff) to sustain cultures, practices and leadership styles which promote and engender supportive working environments and relationships.

The provision and access to confidential support was important. Being able to find a safe, secure, anonymous and easy to access source of support outside of their work environment was valued by participants. To ensure doctors are aware of the available support, signposting information about specialist service such as the PHP could be included in every induction session across the country, and be reinforced by deaneries through Health Education England. Managers and leaders need to facilitate and sanction support for staff when needed.

Participants in this study highlighted that, despite challenging working conditions - long hours, high workloads – they felt better able to cope with these demands and less isolated when they operated within a supportive work culture. Such cultures were

For peer review only

often enabled and enacted by good leadership; consultants with proactive leadership and management styles allowed staff to feel supported by being valued, involved, heard and respected. Consultants who recognise and value the skills, knowledge and experience of team members, invite staff to ask questions and advertise their availability can facilitate learning and empower staff to ask questions or for help, if needed. Such examples of effective management and leadership are supported by evidence which has shown that effective leadership can promote mental health and wellbeing, prevent stress and enable staff to perform at their optimum [26].

Training for leadership styles and strategies which promote team cohesion or which, conversely, reduce the antecedents of toxic work cultures, including bullying, role conflict, role ambiguity, and chaotic and unpredictable work environments [27], is crucial. Employers also need to place greater value on meaningful, inclusive and impactful leadership and management styles which support staff [28].

Findings from this study, reported in a separate paper [Riley et al.2020a under review], revealed the potential for emotional distress when junior doctors work in contrasting cultures where there are no safe spaces to discuss difficult or traumatic work experiences, such as the sudden or violent death of a patient. Feeling supported, permitting time out or time off work when needed, and minimising any potential for isolation and distress in such circumstances can reduce the likelihood of secondary/vicarious trauma (a process of change resulting from the effects of prolonged exposure to human suffering and trauma) [29, 30]. Feeling isolated when distressed is also a risk factor for suicide [31]; providing effective support needs to be an imperative in such contexts.

Participants in this study valued peer support – this is underpinned by evidence attesting the value and importance of social relationships at work in buffering the demands of the job [32]. Belonging, feeling valued, and connectedness are protective factors and can promote positive mental health; for instance, peer support in the workplace has been found to mitigate the negative psychological consequences of adverse events [32]. Our findings highlight the value of working in supportive, cohesive and connected teams and are supported by previous research which found that staff who work in cohesive teams have better mental health, while lack of leadership was associated with poorer mental health [33, 34]. Having continuity in teams, including a named consultant fosters a sense of belonging and produces better patient outcomes [34, 35].

Participants' experiences in this study are supported by the findings of a realist review of interventions and strategies to reduce mental ill health and distress in doctors. This found that leadership and supervision level interventions, supported by the organisation, focusing on relationships, emotional support, and belonging were more likely to promote wellbeing and that fair and equitable learning environments which

For peer review only

balanced positive/negative feedback enabled doctors to thrive [36]. This is underpinned by evidence which found that social connectedness contributes to the reduction of stress levels while social identification (belonging), is beneficial to the psychological health of employees and reduces burnout [32, 36, 37].

Leadership and management can play a key role in shaping group identities and belonging within a team [38]. Trust, defined as, “the extent to which one is willing to ascribe good intentions to, and have confidence in, the words and actions of other people” [39] has been shown to be positively associated with performance, civility and psychological safety [40]. Workforce cultures and environments play an integral role in promoting the mental health of junior doctors through support and creating safe and authentic environments where doctors can excel in their training, and overcome challenges linked to the demands of their job, enabling them to stay well.

CONCLUSION

Our findings show that peer support, effective and supportive leadership and management practices and access to appropriate professional care can help mitigate the negative impact of working conditions and cultures experienced by many junior doctors. Feeling connected, supported and valued by colleagues and consultants acts as an important buffer against emotional distress despite working under challenging working conditions and reduces any potential for isolation.

Authenticity of interactions between senior and junior colleagues was seen as an important aspect of how mental health and well-being are understood and negotiated in the work environment. Normalising vulnerability through disclosure and creating emotion cultures where vulnerability is accepted, provides junior staff greater confidence to be open about factors affecting their own wellbeing and to seek and receive support when needed. Supporting doctors who request time out or time off and facilitating access to support could reduce the potential for isolation in the workplace, reduce stigma-related barriers to help-seeking. Interventions and solutions to minimise distress and support staff are evidenced in existing leadership and peer support, but need to be more consistently practiced across the NHS.

Author Contributions:

RR, CCG, MB, KT, AG, AKT, MvH, LA, JM – substantial contributions to conception and design

RR, FK, CCG, MB, KT, AG, AKT, LA, MvH, acquisition of data, or analysis and interpretation of data; drafting the article or revising it critically for important intellectual content; and final approval of the version to be published.

Acknowledgements: The authors would like to thank all volunteer junior doctor participants who kindly gave their time to share their experiences of working in in the NHS. We also wish to thank members of the PPIE group who provided valuable input throughout the study.

For peer review only

Competing interests: None

Funding: The study was funded by NIHR Research for Patient Benefit (PB-PG-0418-20023)

Department of Health Disclaimer: The views and opinions expressed therein are those of the authors and do not necessarily reflect those of the NIHR, NHS or the Department of Health.

Ethical approval: Ethical approval was granted by the University of Birmingham and Health Research Authority (reference number: 19/HRA/6579)

Data sharing statement: This study has not received ethical approval to share confidential data with any third party other than the study research team.

For peer review only

Tables/Boxes

Box 1: Inclusion and exclusion criteria

INCLUSION CRITERIA	EXCLUSION CRITERIA
Currently a foundation or junior (pre consultant) doctor working in a hospital in the NHS in England/Wales within the last 2 years – this covers any pre-consultant doctor working in the NHS. They do not necessarily have to be on a training contract.	Currently experiencing acute severe mental illness such as psychosis
Experience (within the last 4 years) experience of stress, distress, mental illness, self-harm (e.g. cutting, overdoses), suicidal thoughts, feelings, intent	Currently in receipt of drug and alcohol services (this does not include doctors who currently use alcohol/drugs to self- medicate such as doctors who may be using alcohol/drugs on a regular basis to help them relax but would not be classified as having an addiction problem.)
Has capacity to provide informed consent (this will be assumed given they are healthcare staff)	Actively suicidal or anyone who has made a suicide attempt within the last 6 weeks - to avoid causing any additional psychological harm during a period of acute distress/vulnerability

Table 1: Participant characteristics

Participant characteristics	N=21	%
Gender (female)	16	76

Age (years)		
20-29	10	48
30-39	11	52
Ethnicity		
Asian (other)	2	10
Bangladeshi	1	5
Chinese	1	5
Indian	3	14
White	13	62
White (other)	1	5
Sexual orientation (heterosexual)	15	
Years since qualification		
0-5	10	48
6-10	9	42
11-15	2	10
Specialty		
Emergency medicine	2	10
Medicine (including acute, diabetes/endocrinology, geriatrics)	9	42
Obstetrics and gynaecology	6	28
Paediatrics	2	10
Psychiatry	1	5
Public health	1	5

Box 2: Interview topic guide

Introduction and Background

For peer review only

- Describe general less stressful / more stressful jobs and the difference between these

Work Environment

- Describe main sources of stress in day-to-day working life
- Explore wider sources or stress not already mentioned
- Explore which jobs are more stressful

Impact of work on mental health and wellbeing

- Past, present and future outlook as to how work impacted / may impact mental health and wellbeing?

Preventing / Seeking / Managing help

- Explore management of workload / stress in day-to-day work life
- Discuss help seeking for distressing events
- Explore relationships with colleagues and how concerns are responded to if raised

Experience of help-seeking

- Explore thoughts/feelings of seeking help
- Explore knowledge of available support and protective factors

What could make things better?

- Explore any realistic changes at individual and organisational level and upstream changes

REFERENCES

1. NHS Digital, *NHS Workforce Statistics - January 2020*, in *NHS Hospital & Community Health Service (HCHS) monthly workforce statistics*. 2020, Health and Social Care Information Centre.

2. Brooks, S.K., C. Gerada, and M. Chalder, *Review of literature on the mental health of doctors: Are specialist services needed?* Journal of Mental Health, 2011: p. 1-11.
3. Department of Health, *Mental health and ill health in doctors* 2008, Department of Health: London.
4. Dutheil, F., et al., *Suicide among physicians and health-care workers: A systematic review and meta-analysis.* PloS one, 2019. **14**(12): p. e0226361-e0226361.
5. Milner, A., et al., *Suicide among emergency and protective service workers: A retrospective mortality study in Australia, 2001 to 2012.* Work, 2017. **57**(2): p. 281-287.
6. Schernhammer, E.S.a.C., G.A, *Suicide Rates Among Physicians: A Quantitative and Gender Assessment (Meta-Analysis).* American Journal of Psychiatry, 2004. **161**(12): p. 2295-2302.
7. PHP, *The First Five Years of the NHS Practitioner Health Programme*. 2016.
8. Balme, E., *Doctors need to be supported, not trained in resilience.* BMJ: British Medical Journal, 2015. **351**.
9. McKinley, N., et al., *Resilience in medical doctors: a systematic review.* Postgraduate medical journal, 2019. **95**(1121): p. 140-147.
10. Kinman, G. and K. Teoh, *What could make a difference to the mental health of UK doctors? A review of the research evidence.* 2018.
11. Hayes, B., et al., *What's up doc? A national cross-sectional study of psychological wellbeing of hospital doctors in Ireland.* BMJ Open, 2017. **7**(10).
12. Smith, S.E., et al., *Foundation Year 2 doctors' reasons for leaving UK medicine: an in-depth analysis of decision-making using semistructured interviews.* BMJ Open, 2018. **8**(3).
13. NHS., *Junior doctor morale. Understanding best practice working environments*. 2017, Health Education England.
14. Gunasingam, N., et al., *Reducing stress and burnout in junior doctors: the impact of debriefing sessions.* Postgraduate Medical Journal, 2015. **91**(1074): p. 182-187.
15. Flanagan, E., et al., *Reflection for all healthcare staff: a national evaluation of Schwartz rounds.* Journal of Interprofessional Care, 2020. **34**(1): p. 140-142.
16. Bakker, A.B. and E. Demerouti, *Job demands-resources theory: Taking stock and looking forward.* J Occup Health Psychol, 2017. **22**(3): p. 273-285.
17. van der Doef, M. and S. Maes, *The Job Demand-Control (-Support) model and psychological well-being: A review of 20 years of empirical research.* Work & Stress, 1999. **13**(2): p. 87-114.
18. Charmaz, K., *Constructionism and the grounded theory method.* Handbook of constructionist research, 2008. **1**: p. 397-412.
19. PHP, *https://mysupportt.com/nhs-practitioner-health-programme/.* 2020.
20. Saunders, B., et al., *Saturation in qualitative research: exploring its conceptualization and operationalization.* Quality & quantity, 2018. **52**(4): p. 1893-1907.
21. Braun, V. and V. Clarke, *Using thematic analysis in psychology.* Qualitative research in psychology., 2006. **3**(2): p. 77-101.
22. Charmaz, K., *Constructing grounded theory. A practical guide through qualitative analysis*. 2006, London: Sage.
23. Patton, M., *Enhancing the Quality and Credibility of Qualitative Analysis.* HSR, 1999. **34**(5).
24. Tong, A., P. Sainsbury, and J. Craig, *Consolidated criteria for reporting qualitative research (COREQ): a 32-item checklist for interviews and focus groups.* International journal for quality in health care, 2007. **19**(6): p. 349-357.
25. Spiers, J., et al., *Barriers, facilitators, and survival strategies for GPs seeking treatment for distress: a qualitative study.* Br J Gen Pract, 2017. **67**(663): p. e700-e708.
26. *MIND, Introduction to mentally healthy workplaces* 2020.
27. Samsudin, E.Z., et al., *Organisational antecedents of workplace victimisation: The role of organisational climate, culture, leadership, support, and justice in predicting junior doctors' exposure to bullying at work.* The International Journal of Health Planning and Management, 2020. **35**(1): p. 346-367.

For peer review only

28. King's Fund, *NHS leadership and culture: our position.* 2020.
29. Lanius, R.A., E. Vermetten, and C. Pain, *The impact of early life trauma on health and disease: The hidden epidemic*. 2010: Cambridge, UK.
30. Crumpei, I. and I. Dafinoiu, *The relation of clinical empathy to secondary traumatic stress.* Procedia-Social and Behavioral Sciences, 2012. **33**: p. 438-442.
31. Leigh-Hunt, N., et al., *An overview of systematic reviews on the public health consequences of social isolation and loneliness.* Public Health, 2017. **152**: p. 157-171.
32. van Pelt, F., *Peer support: healthcare professionals supporting each other after adverse medical events.* Quality and Safety in Health Care, 2008. **17**(4): p. 249.
33. Borrill, C., et al., *Team working and effectiveness in health care.* British Journal of Healthcare Management, 2000. **6**(8): p. 364-371.
34. West, M.A. and L. Markiewicz, *Effective team working in health care.* The Oxford handbook of health care management, 2016: p. 231-254.
35. GOV.UK., *NHS staff management and health service quality.* 2019.
36. Carrieri, D., et al., *Optimising strategies to address mental ill-health in doctors and medical students: 'Care Under Pressure' realist review and implementation guidance.* BMC Medicine, 2020. **18**(1): p. 76.
37. West, M. and D. Coia. *Caring for doctors Caring for patients*. 2019.
38. Steffens, N.K., et al., *A Meta-Analytic Review of Social Identification and Health in Organizational Contexts.* Personality and Social Psychology Review, 2016. **21**(4): p. 303-335.
39. Cook, J. and T. Wall, *New work attitude measures of trust, organizational commitment and personal need non-fulfilment.* Journal of Occupational Psychology, 1980. **53**(1): p. 39-52.
40. Leiter, M.P., A. Day, and L. Price, *Attachment styles at work: Measurement, collegial relationships, and burnout.* Burnout Research, 2015. **2**(1): p. 25-35.

For peer review only

Consolidated criteria for reporting qualitative studies (COREQ): 32-item checklist

Developed from:
Tong A, Sainsbury P, Craig J. Consolidated criteria for reporting qualitative research (COREQ): a 32-item checklist for interviews and focus groups. *International Journal for Quality in Health Care*. 2007. Volume 19, Number 6: pp. 349 – 357

No. Item	Guide questions/description	Reported on Page #
**Domain 1: Research team and reflexivity**		
Personal Characteristics		
1. Interviewer/facilitator	Which author/s conducted the interview or focus group?	Page 7
2. Credentials	What were the researcher's credentials? E.g. PhD, MD	Page 7
3. Occupation	What was their occupation at the time of the study?	Page 7
4. Gender	Was the researcher male or female?	Page 7
5. Experience and training	What experience or training did the researcher have?	Page 7
Relationship with participants		
6. Relationship established	Was a relationship established prior to study commencement?	Page 7 .
7. Participant knowledge of the interviewer	What did the participants know about the researcher? e.g. personal goals, reasons for doing the research	Page 7
8. Interviewer characteristics	What characteristics were reported about the inter viewer/facilitator? e.g. Bias, assumptions, reasons and interests in the research topic	Page 7

For peer review only

**Domain 2: study design**		
Theoretical framework		
9. Methodological orientation and Theory	What methodological orientation was stated to underpin the study? e.g. grounded theory, discourse analysis, ethnography, phenomenology, content analysis	Page 7
Participant selection		
10. Sampling	How were participants selected? e.g. purposive, convenience, consecutive, snowball	Page 7
11. Method of approach	How were participants approached? e.g. face-to-face, telephone, mail, email	Page 7
12. Sample size	How many participants were in the study?	Page 9
13. Non-participation	How many people refused to participate or dropped out? Reasons?	None
Setting		
14. Setting of data collection	Where was the data collected? e.g. home, clinic, workplace	Page 8 .
15. Presence of non-participants	Was anyone else present besides the participants and researchers?	No
16. Description of sample	What are the important characteristics of the sample? e.g. demographic data, date	Page 9/19-20
Data collection		
17. Interview guide	Were questions, prompts, guides provided by the authors? Was it pilot tested?	Page 7/20
18. Repeat interviews	Were repeat interviews carried out? If yes, how many?	No, due to lack of resources
19. Audio/visual recording	Did the research use audio or visual recording to collect the data?	Page 7

For peer review only

20. Field notes	Were field notes made during and/or after the interview or focus group?	Yes, Page 8
21. Duration	What was the duration of the inter views or focus group?	Page 8
22. Data saturation	Was data saturation discussed?	Yes, page 8
23. Transcripts returned	Were transcripts returned to participants for comment and/or correction?	No, due to lack of resources
**Domain 3: analysis and findings**		
Data analysis		
24. Number of data coders	How many data coders coded the data?	Page 8
25. Description of the coding tree	Did authors provide a description of the coding tree?	No
26. Derivation of themes	Derived from the data?	Page 8
27. Software	What software, if applicable, was used to manage the data?	Page 8
28. Participant checking	Did participants provide feedback on the findings?	No, due to lack of resources
Reporting		
29. Quotations presented	Were participant quotations presented to illustrate the themes/findings? Was each quotation identified? e.g. participant number	Pages 8-15
30. Data and findings consistent	Was there consistency between the data presented and the findings?	Yes
31. Clarity of major themes	Were major themes clearly presented in the findings?	Yes
32. Clarity of minor themes	Is there a description of diverse cases or discussion of minor themes?	Yes

For peer review only

BMJ Open

Protective factors and sources of support in the workplace as experienced by UK foundation and junior doctors: a qualitative study

Journal:	*BMJ Open*
Manuscript ID	bmjopen-2020-045588.R1
Article Type:	Original research
Date Submitted by the Author:	09-Feb-2021
Complete List of Authors:	Riley, Ruth; University of Birmingham College of Medical and Dental Sciences<br>Kokab, Farina ; University of Birmingham,<br>Buszewicz, Marta; University College London, Research Department of Primary Care and Population Health<br>Gopfert, Anya; Oxford University Hospitals NHS Trust<br>Van Hove, Maria; London School of Hygiene & Tropical Medicine<br>Taylor, Anna<br>Teoh, Kevin; Birkbeck University of London, Department of Organizational Psychology<br>Martin, James; University of Birmingham, Institute of Applied Health Research<br>Appleby, Louis; University of Manchester, Psychiatry & Behavioral Sciences<br>Chew-Graham, Carolyn; University of Keele
**Primary Subject Heading**:	Mental health
Secondary Subject Heading:	Qualitative research, Occupational and environmental medicine, Medical education and training
Keywords:	QUALITATIVE RESEARCH, OCCUPATIONAL & INDUSTRIAL MEDICINE, MENTAL HEALTH, MEDICAL EDUCATION & TRAINING, Suicide & self-harm < PSYCHIATRY

SCHOLARONE™
Manuscripts

I, the Submitting Author has the right to grant and does grant on behalf of all authors of the Work (as defined in the below author licence), an exclusive licence and/or a non-exclusive licence for contributions from authors who are: i) UK Crown employees; ii) where BMJ has agreed a CC-BY licence shall apply, and/or iii) in accordance with the terms applicable for US Federal Government officers or employees acting as part of their official duties; on a worldwide, perpetual, irrevocable, royalty-free basis to BMJ Publishing Group Ltd ("BMJ") its licensees and where the relevant Journal is co-owned by BMJ to the co-owners of the Journal, to publish the Work in this journal and any other BMJ products and to exploit all rights, as set out in our licence.

The Submitting Author accepts and understands that any supply made under these terms is made by BMJ to the Submitting Author unless you are acting as an employee on behalf of your employer or a postgraduate student of an affiliated institution which is paying any applicable article publishing charge ("APC") for Open Access articles. Where the Submitting Author wishes to make the Work available on an Open Access basis (and intends to pay the relevant APC), the terms of reuse of such Open Access shall be governed by a Creative Commons licence – details of these licences and which Creative Commons licence will apply to this Work are set out in our licence referred to above.

Other than as permitted in any relevant BMJ Author's Self Archiving Policies, I confirm this Work has not been accepted for publication elsewhere, is not being considered for publication elsewhere and does not duplicate material already published. I confirm all authors consent to publication of this Work and authorise the granting of this licence.

For peer review only

Protective factors and sources of support in the workplace as experienced by UK foundation and junior doctors: a qualitative study

Ruth Riley < Corresponding author>

Institute of Applied Health Research

University of Birmingham
Birmingham

United Kingdom

r.riley@bham.ac.uk

Tel: + 44 (0) 121 414 8549

Farina Kokab,

Department of Social Work and Social Policy

School of Social Policy

University of Birmingham

Birmingham

United Kingdom

Marta Buszewicz,

Research Department of Primary Care and Population Health

University College London

London

United Kingdom

Anya Gopfert

Oxford University Hospitals Trust

Oxford

For peer review only

United Kingdom

Maria van Hove

London School of Hygiene and Tropical Medicine

London

United Kingdom

Anna Kathryn Taylor

Leeds Institute of Health Sciences

Faculty of Medicine and Health

University of Leeds

Leeds

United Kingdom

Kevin Rui-Han Teoh,

Department of Organizational Psychology
Birkbeck, University of London

London

United Kingdom

James Martin

Institute of Applied Health Research

University of Birmingham
Birmingham

United Kingdom

For peer review only

Louis Appleby

Division of Psychology and Mental Health

School of Medicine

University of Manchester

Manchester

United Kingdom

Carolyn A. Chew-Graham

School of Medicine

Keele University

Keele

United Kingdom

For peer review only

Abstract

Objectives. This paper reports findings identifying foundation and junior doctors' experiences of occupational and psychological protective factors in the workplace and sources of effective support.

Design. Interpretative, inductive, qualitative study involving in-depth interviews with 21 junior doctor participants. The interviews were audio-recorded, transcribed, anonymised and imported into NVivo11 to facilitate data management. Data were analysed using a thematic analysis employing the constant comparative method.

Setting. National Health Service in the United Kingdom.

Participants. Participants were recruited from junior doctors through social media (for example, the BMA junior doctors' Facebook group, Twitter and the mental health research charity websites). A purposive sample of 16 female and 5 male, ethnically diverse, from a range of specialities, across the UK. Junior doctor participants self-identified as having stress, distress, anxiety, depression and suicidal thoughts or having attempted to kill themselves.

Results. Analysis identified three main themes, with corresponding subthemes relating to protective work factors and facilitators of support: 1. Support from work colleagues – help with managing workloads and emotional support. 2. Supportive leadership strategies, including feeling valued and accepted, trust and communication, supportive learning environments, challenging stigma and normalising vulnerability 3. Access to professional support – counselling, CBT and medication through General Practitioners, specialist support services for doctors, and private therapy.

Conclusions. Findings show that supportive leadership, effective management practices, peer support, and access to appropriate professional support can help mitigate the negative impact of working conditions and cultures experienced by junior doctors. Feeling connected, supported and valued by colleagues and consultants acts as an important buffer against emotional distress despite working under challenging working conditions.

Keywords: Qualitative, Hospital Staff, Occupational Stress, Mental Health, Suicide, Psychological Support

Word count: 3889

For peer review only

Strengths and limitations of this study

- Few qualitative studies have explored work-related psychological distress in junior doctors; we therefore employed in-depth interviews with 21 junior doctors from across the United Kingdom to explore this distress

- The sample included five male participants compared with 16 female participants which may be a limitation in this study; however, the purposive sampling, of participants, ensured the sample was varied in terms of ethnicity, number of years in training, speciality, and geographical location. .

- Participants self-identified as having chronic stress and/or mental health problems, including anxiety and depression; therefore the findings may not be generalizable to the general population of junior doctors.

- Semi-structured interviews generated rich qualitative data and an iterative process of concurrent data collection and constant comparative analysis facilitated the simultaneous exploration, refinement and enrichment of key themes.

INTRODUCTION

There are currently 115,376 doctors working in the NHS, almost half of whom (56,404) are termed 'junior doctors', which includes doctors in specialist training or at pre-Consultant grade, and Foundation Year doctors [1]. Previous research indicates that doctors are vulnerable to chronic stress, anxiety, depression and burnout [2, 3]. Severe depression has also been identified as a risk factor for suicide [4, 5]. High rates of suicide have frequently been reported among doctors, but with considerable heterogeneity over time and between countries [6]. Despite mixed findings about suicide rates internationally, there are concerns over suicidal ideation and behaviour in doctors, particularly women [7, 8]. International research indicates that psychological distress (such as depression or anxiety) is higher in female compared to male doctors [9].

The Practitioner Health Programme (PHP) in London, an England-wide specialist mental health, drug and alcohol service for doctors and dentists, reports that two-thirds of those presenting are women, with higher attendance rates among young female doctors [10]. It is uncertain whether this is representative of the general population of doctors or reflects gender differences in help-seeking behaviour.

Most previous research into distress and suicide in junior doctors reports quantitative, retrospective studies, with the focus on the individual risk factors (particularly emphasising the need for 'resilience'), [11, 12] and overlooking the wider systemic, organisational and cultural sources of distress among this population of health professionals. Focusing on individual risk factors and interventions are likely to detract from considering solutions or interventions targeting the learning environment, organisational culture and systemic factors [13, 14]. To our knowledge, no previous study has qualitatively examined how junior doctors view their working conditions (e.g. rotas, spaces to rest, eat and sleep when on-call) and work cultures (relationships, support, job control), and the factors (e.g. debriefs following critical incidents,

For peer review only

policies, available support, management practices) which may protect them from psychological distress or offer them support.

Junior doctors face specific pressures related to their professional stage and development. In a nationwide survey of junior doctors in Ireland, stress, anxiety and depression were highest among junior doctors as compared to consultants [15]. A study exploring junior doctors' reasons for leaving medicine in the UK cited lack of support, mentorship or formal training, loneliness and bullying [16]. A qualitative study undertaken in Australia found that lack of support from colleagues, working beyond one's perceived abilities and neglecting self-care were key contributors to burnout and reduced wellbeing among junior doctors [17]. Doctors under investigation are also at higher risk of suicide and psychological distress [18, 19]; those in receipt or facing threats of complaints experience elevated anxiety, depression and suicidal thoughts compared to colleagues who face no complaints [20, 21].

A lack of support, mental health stigma, and toxic work cultures, characterised by bullying and a blame and shame culture were identified as a key source of distress among junior doctors, in a separate paper linked to this study [Riley et al. 2020 – under review BMJ Open]. Similarly, feeling undervalued, unsupported or having reduced autonomy were key factors affecting low morale among junior doctors [22]; this highlights the impact of working conditions and work culture on junior doctors and the potential value of providing effective support and supervision.

There is substantial evidence linking longer working hours with mental ill health and reduced patient safety; junior doctors who worked over 55 hours a week were more than twice as likely to report a common mental health problem [23] and increased likelihood of making a medical error [24]. This highlights the importance of providing suitable facilities for sleep, rest and social spaces, and to facilitate social interaction and access to collegial support [25].

Evidence has found that junior doctors valued the emotional support derived from attending debriefing sessions following adverse events, although its effectiveness in reducing burnout was equivocal [26]. Workplace interventions such as Schwartz rounds, which provide permission and a safe space for healthcare staff to talk about the range of feelings and challenges which arise during their work while also fostering connectedness to others, were valued by staff [27]. Although numerous workplace health theories [28, 29] identify effective support as a core antecedent to positive mental health and as a potential buffer against challenging work environments, how this might manifest amongst junior doctors is unclear. Identifying such patterns or contextual factors is crucial in developing targeted improvements or interventions that can reduce the risk of mental ill health and psychological distress from the outset.

Occupational or work-related stress are also compounded by barriers to help-seeking which include difficulties accessing healthcare due to working hours, perceived stigma, concerns about loss of confidentiality, therapeutic nihilism (a cynicism about the effectiveness and availability of existing interventions for mental ill health) and pressure to continue working while unwell, due to staff shortages and concerns about letting colleagues down [2, 9, 30, 31].

Facilitators included the availability of confidential, specialised services for doctors, emotional and practical support such as finding cover for shifts [30]. Shanafelt et al. [32] recommend

For peer review only

various interventions (e.g. positive role models, training of group and teams, inter-professional support groups) to facilitate culture change. Promoting wellbeing by providing psychological safety and positive learning environments may reduce learning anxiety among junior doctors [33].

This qualitative study is part of a wider mixed-methods study examining the psychological, cultural and occupational contexts associated with reduced psychological wellbeing in junior doctors, and protective factors which may mitigate against this. This paper reports the qualitative findings focussed on the protective factors and sources of effective support in the workplace as experienced by junior doctors.

METHODS

Study design and setting

The study methodology was underpinned by a constructivist grounded theory approach [34], using qualitative methods, with semi-structured interviews to explore junior doctors' perspectives and experiences of stress and distress. The study setting was the NHS in the UK.

Sampling and Recruitment

We used a range of recruitment methods including advertisements on social media (for example, the junior doctors' Facebook group, Twitter and the mental health research charity websites). In addition, information about the study was circulated through the Practitioner Health Programme, a specialised mental health and drug and alcohol service dedicated to doctors and dentists [35].

Potential participants were asked to express their interest by contacting members of the study team who then provided further information about the study.

The research team employed purposive sampling from the responding junior doctors, to ensure maximum variation taking account of the following characteristics: gender, age, ethnicity, sexuality, geographical location, different grades/duration of General Medical Council registration, medical speciality, disclosure of a mental health diagnosis, individuals who reported self-harm behaviour, had thoughts of suicide or had attempted to kill themselves.

The eligibility criteria used are listed in Box 1.

Junior doctors interested in taking part were sent a reply slip and consent form electronically and asked to sign the consent form prior to the interview, before returning it to the study researcher. Face-to-face, telephone or 'virtual platform' interviews were arranged at a time convenient to each participant. They were given the opportunity to raise questions prior to being interviewed and during the interview.

At the end of each interview, the researcher informed the participant about the next steps, how their data would be used, and checked on their wellbeing prior to leaving the interview setting.

For peer review only

A risk protocol was used to ensure appropriate support was provided to participants (and the researcher) in the event of the disclosure of significant distress or suicidal ideation.

Data collection

A topic guide (see box 2) was developed by the research team to generate discussion in the semi-structured interviews. The topic guide was informed by the existing literature, input from junior doctors on the study team, and patient and public involvement (PPI) input (see below), and modified iteratively as data collection and analysis progressed. The topic guide aimed to capture participants' views, experiences, feelings and beliefs about working conditions and cultures which were perceived to be stressful or distressing, and factors which were perceived/felt to be protective.

One-to-one interviews were conducted face-to-face (n=7), by telephone (n=13) or using a digital virtual platform (n=1) and digitally recorded with consent. The in-depth interviews were conducted by two researchers (FK, RR), both female social and behavioural scientists with qualitative methods expertise. The recorded interviews were transcribed verbatim and checked for accuracy of transcription by the study researcher before analysis. All transcripts were anonymised before discussion within the wider research team. Reflexive notes were recorded throughout the process. Recruitment and data collection was continued until data saturation was judged to have been achieved [36].

Data analysis

Analysis began with data collection, and was iterative **and inductive,** employing the constant comparative method [37, 38] until theoretical data saturation was achieved, such that no new analytic categories emerged [36].

The research team included three junior doctors (AG, AKT, MvH), two academic general practitioners (CCG, MB), one occupational health psychologist (KT) and two social scientists (RR and FK). FK coded the data set; RR coded a sub-sample and contributed to the organisation of themes. The multidisciplinary team provided commentary on transcripts to generate and refine codes and thematic categories and provide researcher triangulation, thereby increasing the credibility of the research findings [39]. Data were managed using NVivo 11. The study is reported in line with the consolidated criteria for reporting qualitative research (COREQ) [40].

Patient and Public Involvement

There were three junior doctors on the research team, all of whom consulted with colleagues about the initial research idea. It was felt important that the research focus needed to explore working conditions and cultures, rather than focusing the gaze inward or on individuals. Five junior doctors provided feedback on the initial funding application, and four of these gave feedback on the protocol, topic guide and participant facing documents. Junior doctors have been actively involved in publicising the study to junior doctors and we are working on a dissemination strategy with junior doctors outside the research team. Due to the time constraints of junior doctors, PPIE members were consulted via email and telephone.

FINDINGS

For peer review only

Twenty-one interviews were conducted, lasting between 43 and 103 min (mean= 65 min), between November 2019 and May 2020. The demographic and professional characteristics of participants are included in Table 1. Analysis of the interview transcripts and field notes identified three main themes, with corresponding subthemes relating to protective work factors and facilitators of support: 1. Support from work colleagues, including help with managing workloads and emotional support. 2. Supportive leadership strategies, including feeling valued and accepted, trust and communication, supportive learning environments and challenging stigma and normalising vulnerability 3. Access to professional support, including Cognitive Behavioural Therapy (CBT), counselling and medication through General Practitioner and specialist support services for doctors, and accessing private counselling/therapy.

SUPPORT FROM WORK COLLEAGUES

Many participants emphasised the importance of working in protective and supportive work cultures, characterised by good interpersonal relationships, a strong team morale where there was a shared responsibility for workloads, and where staff felt supported with the clinical components of their work and the emotional impact of their job. Crucially, participants highlighted the interdependence between collegial support, supportive work cultures and their emotional wellbeing and ability to cope with the demands of their job.

a) Help with managing workloads

Participants identified the benefits of having mutually supportive interpersonal connections at work where there was a willingness to share responsibilities, offer practical support, helping with workloads or specific tasks:

"*I've made good relationships… [name of speciality] was actually having a very, very nice group of colleagues on the whole and supporting each other. Like offering help if we're struggling, if someone's struggling with a task then I would offer help and vice versa.*" (JD12, male)

The following participant highlighted that nurses offered support by undertaking tasks which free up time for junior doctors to focus on clinical work: Spending time with supportive staff could alleviate some of the burden and stress that junior doctors may be experiencing, especially in new and unfamiliar roles.

"My relationships with my colleagues, both nurses and doctors, are incredible. I couldn't say a bad word about any of them. They have been so wonderfully supportive. The nursing staff have carried me in every job I've been on. They've carried me because they're the constant and they're there all the time." (JD17, female)

b) Emotional support

Participants iterated the importance of peer support and feeling emotionally supported within the team, for example, in response to critical incidents, or being bullied by a colleague::

"*A couple of weeks ago my friend was having a terrible time with a registrar who was being hideous to her and so, you know we kind of helped support her through that and empathise with her and you know I think that's really helpful.*" (JD20, female)

For peer review only

The following participant referred to the '*in it together'* team spirit and camaraderie which acted as an emotional buffer when working under challenging working conditions:

"*We were all in it together and actually, the working culture is really, really good … even in the rubbish conditions that we're working in, it's the people that have made it worthwhile and they've helped me cope with things, so if I'm having a bad day or I'm a bit down, actually it's the camaraderie of working with midwives, other doctors, nurses, pharmacists, porters that have made it quite a happy thing to do really*." (JD09. female)

Many participants had contrasting experiences on different rotations; the following participant described her experience of being in a supportive work culture in the context of a critical incident, which she compared with the 'blame culture' experienced on a previous rotation:

"*In the unit I'm in at the moment, it's a lot more about learning rather than blaming. I think you feel safer then to express opinions or to discuss cases. You want to find out who was involved in order to be able to support them and that's the feeling that you get from the senior midwives and the consultants you work with… The unit that I was in before was really challenging in terms of feeling like you wouldn't be supported, even if you'd done nothing wrong."* (JD13, female)

Another participant reported feeling '*emotionally healthy'* which she attributed to the supportive work culture. She contrasted this with a previous rotation where the cultural norm was to disengage with challenging emotions, leaving staff with unprocessed trauma:

*"I feel really emotionally healthy now. Cause I'm in such a supportive culture. But I look now particularly at [name of specialty] culture as a lot of people who, with a lot of unprocessed trauma. Huge amounts of unprocessed trauma. Amazing really that whole profession can be so in denial of the emotional impact of their job." (*JD23, female)

The value of working in a supportive work culture was further highlighted by the following participant who recollected that having greater support from colleagues in her previous post would have reduced her sense of anxiety:

"I think if I'd have had that [informal support from colleagues] support in first year of someone who could listen, maybe I wouldn't have been so anxious about everything." (JD09, female)

The following participant suggested that, despite the potential stigma associated with mental illness, peer-support was often the first line of support for colleagues:

"I do think peer support is very important and I think that's probably the first line for a lot of people, like even with some stigma and embarrassment about mental health, I think most people would usually turn to a colleague first." (JD16, female)

SUPPORTIVE LEADERSHIP STRATEGIES

a) Feeling valued and accepted

For peer review only

This reoccurring theme refers to supportive and proactive leadership and management approaches to providing support to the team and promoting positive mental health. Participants relayed the importance of feeling valued, included and accepted as part of the team by the consultants. This included receiving recognition and praise for their efforts and contributions, particularly when working in challenging, highly pressurised environments and working long shifts:

'… it [first job] was over 12 hours every single day, you know I think I started to cry because just at least someone was acknowledging the fact that we were doing 12, 13 hour days every day … it was extremely hard but…there was a very good group of consultants who just acknowledged that it was hard, there was not much they could do about it, they had to work hard as well but they were very, very, very supportive of that… they [consultants] just knew what your name was, they knew your role, you felt valued there." (JD01, male)

"*…it makes such a difference and then also like being empowered to be of good patient care, so you know not being shouted at if you've taken half an hour to see a patient and actually you know being recognised, 'Oh you did a really thorough job actually, well done, good for you' and kind of you know just actually some recognition of what you're doing. Yeah, that's what is good, I guess.*" (JD20, female)

This participant emphasised the importance of feeling valued by consultants or patients and its relationship to job satisfaction and confidence:

"…when you receive some good feedback, you can really get a bit of a buzz and a bit of a high from that, either from a patient or from a colleague when they said, you know, 'you've really made a difference today' or 'you've really helped me today' or 'you've made me feel better, thank you'. Whenever you get something like that, which doesn't happen all the time by any means, but when you get a bit of feedback, it makes you realise why you're doing it and I guess all those feelings of anxiety and the feeling of not being good enough, which is a big thing for me. So, if somebody's appreciative, I think that can brighten your day really and make you feel like you're worth something." (JD09, female)

b) Trust and communication

A few participants recalled the value of consultants who were proactive in their support of staff, openly inviting them to discuss concerns, ask questions or to enquire about their work and wellbeing. Establishing these open channels of communication and being approachable made it easier for participants to seek support or advice when needed:

"*Whereas, the two consultants that I go to for that sort of support always made it clear that they were open to talking about anything that was bothering me. They continued to keep that relationship open, even when I wasn't working with them anymore. It was just the odd touch base email like, 'How are things going? What are you up to? Let's have a coffee one day'. It was that kind of thing. You're made to feel a bit less like you're onerous or that you're bothering them when you ask them about something.*" (JD13, female)

Such was the importance of supportive relationship with consultants, a few participants had maintained those connections after moving to a new post:

For peer review only

"*I've got a couple of people that I go back to when I need support ….and I know will speak to them both but I will always go back to those few people that I trust and I know."* (JD19, female)

c) Supportive learning environments

Participants reflected on work cultures where there was a shared commitment to team learning. Such an ethos enabled people to practice without fear of reprisal, to reflect on their work, and to view errors as opportunities for learning and improvement:

"*My ST1 [*Specialist Training year one*] year was extremely enjoyable, because again it was a very supportive place to work and they encouraged you to ask questions and didn't criticise and there was a real sort of no blame culture*" (JD01, male)

Some participants suggested that cohesiveness within teams could be enabled through multidisciplinary training opportunities. These events facilitated a shared understanding of different roles within the team and awareness of the challenges and concerns faced by different professional groups:

"*Where I am at the moment is really good. The current unit I'm in has a lot of multi-professional training that isn't necessarily clinical. We have multi-professional leadership days where the senior junior doctors at ST5 level plus and the senior midwives all get together to do the training together. I think that really helps because we understood their role and their concerns when they're coordinating a unit and they understand that we can't really do everything"* (JD13, female)

d) Challenging stigma and normalising vulnerability

A number of participants emphasised the role of consultants and supervisors in de-stigmatising mental ill health by sharing or disclosing their vulnerability or experiences of mental ill health to their staff; participants report that this created a more open and authentic work culture where staff also felt safe to disclose their vulnerability:

"We were working a Sunday together and I said, 'I haven't seen you in a while.' She said, 'No, I have been off for about four months.' I said, 'Are you just coming back?' She said, 'Yeah, I was off because I had a mental breakdown. One day, I just realised that I was really, really unwell and I just had to go home and I didn't return for a number of months until I got myself sorted out.' My point was just her talking about that, I think, does wonders because she's a woman who has been there forever. She's a senior consultant. Just telling other people, 'Yeah, I've gone through the struggles. I need medication for this, that and the other. It's okay. I'm still here functioning and it's fine'. I think people need to be more open about that," (JD03, male)

"I think what really needs to change is that I think that more senior doctors need to be open about their struggles and their issues in training because I think there's a perception that you need to be invulnerable among more junior trainees in particular, which I think as people gradually have breakdowns, they're robbed of that illusion. Yes, yes, I think that kind of cultural change needs to kind of happen." (JD07, female)

For peer review only

ACCESS TO PROFESSIONAL SUPPORT

Few participants reported seeking formalised help from within the workplace (e.g. occupational health) but some study participants had sought professional help from counsellors, therapists, general practitioners (GPs) and specialist services for doctors, outside the workplace. The following participant was prescribed antidepressants by her GP to manage her distress:

"…I was in tears and nervous …I kept on saying, 'I don't feel like this is going to ever change. I don't feel confident,' and then I started antidepressants and incredibly, within about two weeks, I was sleeping. I felt better and I felt calm. I realised just how sick I had been." (JD08, female)

Accessing external support was not without barriers. The following participant highlights the significant delay in accessing a counselling referral through their GP:

"My GP referred me for local support and it took them a year to contact me…I mean, it's just the NHS, that's how it is…and even then, it would be one o'clock, two o'clock, it would be nigh on impossible to actually access," (JD19, female)

Having access to services tailored to the needs of junior doctors was important for participants:

"*Because you know that their [*specialist service*] whole patient group are doctors and they've got such expertise of talking to doctors and professionals with the same issues, there is none of that having to make an excuse for yourself. We're so often held up in society as a really responsible, clever and respected group that you can be vulnerable in the PHP. That was really, really validating.*" (JD08, female)

The following participant found out about the Practitioner Health Programme (PHP) by chance (a family member found a PHP leaflet in a waiting room); she was concerned about being referred to a psychiatrist in her local trust because she was planning on undertaking a rotation there in the future:

"*There is the Practitioner Health Programme… I've been seeing a GP, that lives down the road from me, who's absolutely amazing…I feel like that's a really good setup…I didn't feel comfortable being treated in the mental health trust here and also, I want to look at working in that mental health trust. Being able to move my care completely away from that is very important. I didn't know that was there at the beginning. That's when I ran into some of my problems and when I wasn't seeking the help I needed because I didn't know that I could have anonymity.*" (JD22, female)

Due to concerns about the potential loss of confidentiality, the following participant self-referred to a private clinic to seek the formal support they needed:

"*I went to a private mental health clinic and I referred myself, I phoned up and (? they) said well the Consultant probably would take a self-referral especially if you're a Doctor, so I wrote my own referral letter and I went privately to the Psychiatrist across the road from the hospital*

For peer review only

and he got me some treatment which was mainly in the form of counselling … And that changed everything, you know that turned everything around." (JD15, female)

A few participants felt it would be beneficial if junior doctors had access to monthly therapy or supervision sessions, either group work or one-to-one, a safe space to share and process the emotional impact of the job and seek support:

"If, as junior doctors, you saw a therapist once a month... if we made it universal (which is very easy to say) that for one hour a month, everyone had to see someone to talk to, I think that would be a good way of moving forward. If there was something that was really serious, it's a safe space and we can talk about it. If it needs to be escalated or if any sort of treatment needs to be started in whichever way that might be, go for it." (JD03, male)

"*…sometimes lots of stuff coming up and yet there's absolutely nothing. For me I feel like, every doctor should have some form of emotional supervision*." (JD23, female)

DISCUSSION

The findings highlight the value of support from work colleagues and work cultures engendered by camaraderie and connectedness with colleagues. The emotional support offered by colleagues often enabled junior doctors to manage the challenges of the job and was considered to be potentially protective against mental ill health. Participants felt more able to cope with the challenges of their work when their managers and leaders valued staff, offering clinical and emotional support and creating positive learning environments which reduce learning anxiety by providing psychological safety [32, 33]**. Positive learning cultures, characterised by social support, validation of success, and positive affirmation have been found to be protective against burnout [41]. These findings also support** the job-demand-resources theory [28]; namely, that key resources such as support from colleagues, quality of the relationships with supervisors/consultants and feeling valued can buffer the impact of those demands related to their work (long hours, high workload) and consequently protect against burnout.

Our findings also highlight the role of consultants and other senior staff in de-stigmatising mental ill health by acknowledging and sharing their own vulnerability and creating a safe and authentic work culture which enabled staff to seek support. Promoting emotionally open cultures which facilitate disclosure by de-stigmatising mental ill health and allowing for vulnerability, empowers staff to seek support and could reduce the sense of isolation and shame often experienced by junior doctors [30]. Challenging and addressing stigma in medical school and beyond is a crucial stepping stone in enabling doctors to seek help without fear of reprisal or shame [9, 42, 43].

These findings highlight the positive impact of harnessing existing workforce capital (i.e. the skills and training of staff) to sustain cultures, practices and leadership styles which promote and engender supportive working environments and relationships. Participants in this study valued the support provided by work colleagues – this is underpinned by evidence attesting the value and importance of social relationships at work in buffering the demands of the job [44]. Belonging, feeling valued, and connectedness are protective factors and can promote

For peer review only

positive mental health and a positive professional identity; for instance, peer/colleague support in the workplace has been found to mitigate the negative psychological consequences of adverse events [44, 45]. Authors recommend checking-in with colleagues, listening, reflecting, reframing, sense-making, coping, closing, and resources/referrals which can reduce the isolation and sense of shame experienced by colleagues [46].

Our findings highlight the value of working in supportive, cohesive and connected teams and are supported by previous research which found that staff who work in cohesive teams have better mental health, while lack of leadership was associated with poorer mental health [47, 48]. Having continuity in teams, including a named consultant fosters a sense of belonging and produces better patient outcomes [48, 49].

Participants in this study highlighted that, despite challenging working conditions - long hours, high workloads – they felt better able to cope with these demands and less isolated when they operated within a supportive work culture. Such cultures were often enabled and enacted by good leadership; consultants with proactive leadership and management styles allowed staff to feel supported by being valued, involved, heard and respected. Consultants who recognise and value the skills, knowledge and experience of team members, invite staff to ask questions and advertise their availability, can facilitate learning and empower staff to ask questions or for help, if needed. Such examples of effective management and leadership are supported by evidence which has shown that effective leadership can promote mental health and wellbeing, prevent stress and enable staff to perform at their optimum [50].

Training for leadership styles and strategies which promote team cohesion or which, conversely, reduce the antecedents of toxic work cultures, including bullying, role conflict, role ambiguity, and chaotic and unpredictable work environments [51], is crucial. Employers also need to place greater value on meaningful, inclusive and impactful leadership and management styles which support staff [52]. However, NHS policy makers must also ensure that efforts are made to deliver sustainable workforce cultures which avoid an over-dependency on individual leaders. A whole systems approach to prevention and provision will need to be adopted to promote healthy workplaces for all NHS staff. This includes addressing systemic causes of stress such as workforce shortages, rota gaps, and improving physical working conditions, while also promoting work cultures which enable help-seeking and foster belonging and connectedness [13].

Working in effective and supportive teams requires staff to have shared objectives, values and continuity, necessitating planning and changes to rotas which can help to ensure junior doctors feel part of a team [53]. There is substantial evidence that working in teams which lack continuity in staff or shared objectives is detrimental to the mental health and wellbeing of staff and impacts negatively on patient care and safety [48, 49]. Junior doctors need to feel valued, capitalising on existing support infrastructure and assets (i.e. teams, supervisors, colleagues) to provide clinical and psychological support (e.g. interprofessional debriefs for processing and validating feelings). Organisations will need to provide the training and coaching to develop effective and cohesive teams, while also meeting recommendations set out in existing guidance to ensure the clinical, learning, training and emotional needs of doctors are met [54].

For peer review only

Findings from this study, reported in a sister paper [Riley et al.2020a in press], revealed the potential for emotional distress when junior doctors work in contrasting cultures where there are no safe spaces to discuss difficult or traumatic work experiences, such as the sudden or violent death of a patient. Feeling supported, permitting time out or time off work when needed, and minimising any potential for isolation and distress in such circumstances can reduce the likelihood of secondary/vicarious trauma (a process of change resulting from the effects of prolonged exposure to human suffering and trauma) [55, 56]. Feeling isolated when distressed is also a risk factor for suicide [57]; providing effective support needs to be an imperative in such contexts.

The provision and access to confidential support was important to participants and evidenced by previous research [2, 30]. Being able to find a safe, secure, anonymous and easy to access source of support outside of their work environment was greatly valued. To ensure doctors are aware of the available support, signposting information about specialist services such as the Practitioner Health Programme could be included in every induction session across the country, and be reinforced by specialist training schools through Health Education England. Some participants however preferred accessing a trusted GP. Managers and leaders therefore need to facilitate and sanction appropriate support for staff when needed.

Participants' experiences in this study are supported by the findings of a realist review of interventions and strategies to reduce mental ill health and distress in doctors. This found that leadership and supervision level interventions, supported by the organisation, focusing on relationships, emotional support, and belonging were more likely to promote wellbeing and that fair and equitable learning environments which balanced positive/negative feedback enabled doctors to thrive [58]. This is underpinned by evidence which found that social connectedness contributes to the reduction of stress levels while social identification (belonging), is beneficial to the psychological health of employees and reduces burnout [44, 53, 58].

Leadership and management can play a key role in shaping group identities and belonging within a team [59]. Trust, defined as, "the extent to which one is willing to ascribe good intentions to, and have confidence in, the words and actions of other people" [60] has been shown to be positively associated with performance, civility and psychological safety [61]. Workforce cultures and environments play an integral role in promoting the mental health of junior doctors through support and creating safe and authentic environments where doctors can excel in their training, and overcome challenges linked to the demands of their job, enabling them to stay well.

This study has some limitations. There is a notable disparity in gender, with a higher proportion of female doctors participating. We received higher expressions of interest from female (n=37) participants compared to male (n=22), with a higher proportion of female participants then agreeing to participate in an interview. The interest in this study and increased willingness amongst female participants to come forward and talk about their experiences may reflect evidence indicating that female doctors are more likely to experience distress, with increased rates of suicide evidenced in young female doctors. The higher proportion of female participants may also reflect gendered help-seeking behaviour for mental ill health, evidenced also in the wider population [62]. Another potential limitation relates to the lower number of

For peer review only - http://bmjopen.bmj.com/site/about/guidelines.xhtml

participants who reported having had suicidal thoughts (n=5) and participants who disclosed that they had made self-harm attempts (n=2).

CONCLUSION

Our findings show that support from colleagues, effective and supportive leadership and management practices and access to appropriate professional care can help mitigate the negative impact of working conditions and cultures experienced by many junior doctors. Feeling connected, supported and valued by colleagues and consultants acts as an important buffer against emotional distress despite working under challenging working conditions and reduces any potential for isolation.

Authenticity of interactions between senior and junior colleagues was seen as an important aspect of how mental health and well-being are understood and negotiated in the work environment. Normalising vulnerability through disclosure and creating emotionally open cultures where vulnerability is accepted and understood, allows junior staff greater confidence to be open about factors affecting their own wellbeing and to seek and receive support when needed. Supporting doctors who request time out or time off and facilitating access to support could reduce the potential for isolation in the workplace and reduce stigma-related barriers to help-seeking. Examples of effective interventions and solutions to minimise distress and support staff are evidenced in existing leadership and collegial support, but need to be more consistently practiced across the NHS.

Author Contributions:

RR, CCG, MB, KT, AG, AKT, MvH, LA, JM – substantial contributions to conception and design

RR, FK, CCG, MB, KT, AG, AKT, LA, MvH, acquisition of data, or analysis and interpretation of data; drafting the article or revising it critically for important intellectual content; and final approval of the version to be published.

Acknowledgements: The authors would like to thank all volunteer junior doctor participants who kindly gave their time to share their experiences of working in in the NHS. We also wish to thank members of the PPIE group who provided valuable input throughout the study.

Competing interests: None

Funding: The study was funded by NIHR Research for Patient Benefit (PB-PG-0418-20023)

Department of Health Disclaimer: The views and opinions expressed therein are those of the authors and do not necessarily reflect those of the NIHR, NHS or the Department of Health.

Ethical approval: Ethical approval was granted by the University of Birmingham and Health Research Authority (reference number: 19/HRA/6579)

For peer review only

Data sharing statement: This study has not received ethical approval to share confidential data with any third party other than the study research team.

Tables/Boxes

Box 1: Inclusion and exclusion criteria

**INCLUSION CRITERIA**	**EXCLUSION CRITERIA**
Currently a foundation or junior (pre consultant) doctor working in a hospital in the NHS in England/Wales within the last 2 years – this covers any pre-consultant doctor working in the NHS. They do not necessarily have to be on a training contract.	Currently experiencing acute severe mental illness such as psychosis
Experience (within the last 4 years) experience of stress, distress, mental illness, self-harm (e.g. cutting, overdoses), suicidal thoughts, feelings, intent	Currently in receipt of drug and alcohol services (this does not include doctors who currently use alcohol/drugs to self- medicate such as doctors who may be using alcohol/drugs on a regular basis to help them relax but would not be classified as having an addiction problem.)
Has capacity to provide informed consent (this will be assumed given they are healthcare staff)	Actively suicidal or anyone who has made a suicide attempt within the last 6 weeks - to avoid causing any additional psychological harm during a period of acute distress/vulnerability

Table 1: Participant characteristics

**Participant characteristics**	**N=21**	**%**
Gender (female)	16	76
Age (years)		
20-29	10	48
30-39	11	52
Ethnicity		
Asian (other)	2	10

For peer review only - http://bmjopen.bmj.com/site/about/guidelines.xhtml

Bangladeshi	1	5
Chinese	1	5
Indian	3	14
White	13	62
White (other)	1	5
Sexual orientation (heterosexual)	15	
Years since qualification		
0-5	10	48
6-10	9	42
11-15	2	10
Specialty		
Emergency medicine	2	10
Medicine (including acute, diabetes/endocrinology, geriatrics)	9	42
Obstetrics and gynaecology	6	28
Paediatrics	2	10
Psychiatry	1	5
Public health	1	5

Box 2: Interview topic guide

Introduction and Background

- Describe general less stressful / more stressful jobs and the difference between these

Work Environment

- Describe main sources of stress in day-to-day working life
- Explore wider sources or stress not already mentioned
- Explore which jobs are more stressful

For peer review only

Impact of work on mental health and wellbeing

- Past, present and future outlook as to how work impacted / may impact mental health and wellbeing?

Preventing / Seeking / Managing help

- Explore management of workload / stress in day-to-day work life
- Discuss help seeking for distressing events
- Explore relationships with colleagues and how concerns are responded to if raised

Experience of help-seeking

- Explore thoughts/feelings of seeking help
- Explore knowledge of available support and protective factors

What could make things better?

- Explore any realistic changes at individual and organisational level and upstream changes

REFERENCES

1. NHS Digital, *NHS Workforce Statistics - January 2020*, in *NHS Hospital & Community Health Service (HCHS) monthly workforce statistics*. 2020, Health and Social Care Information Centre.
2. Brooks, S.K., C. Gerada, and M. Chalder, *Review of literature on the mental health of doctors: Are specialist services needed?* Journal of Mental Health, 2011: p. 1-11.
3. Department of Health, *Mental health and ill health in doctors* 2008, Department of Health: London.
4. Hawton, K., et al., *Risk factors for suicide in individuals with depression: A systematic review.* Journal of Affective Disorders, 2013. **147**(1): p. 17-28.
5. Menon, N.K., et al., *Association of Physician Burnout With Suicidal Ideation and Medical Errors.* JAMA Network Open, 2020. **3**(12): p. e2028780-e2028780.
6. Dutheil, F., et al., *Suicide among physicians and health-care workers: A systematic review and meta-analysis.* PloS one, 2019. **14**(12): p. e0226361-e0226361.
7. Milner, A., et al., *Suicide among emergency and protective service workers: A retrospective mortality study in Australia, 2001 to 2012.* Work, 2017. **57**(2): p. 281-287.
8. Schernhammer, E.S. and G.A. Colditz, *Suicide rates among physicians: a quantitative and gender assessment (meta-analysis).* American Journal of Psychiatry, 2004. **161**(12): p. 2295-2302.

9. Beyondblue, *The Mental Health of Doctors: A Systematic Literature Review: Executive Summary*. 2010.
10. PHP, *The First Five Years of the NHS Practitioner Health Programme*. 2016.
11. Balme, E., *Doctors need to be supported, not trained in resilience.* BMJ: British Medical Journal, 2015. **351**.
12. McKinley, N., et al., *Resilience in medical doctors: a systematic review.* Postgraduate medical journal, 2019. **95**(1121): p. 140-147.
13. Kinman, G. and K. Teoh, *What could make a difference to the mental health of UK doctors? A review of the research evidence.* 2018.
14. Dyrbye, L. and T. Shanafelt, *A narrative review on burnout experienced by medical students and residents.* Med Educ, 2016. **50**(1): p. 132-49.
15. Hayes, B., et al., *What's up doc? A national cross-sectional study of psychological wellbeing of hospital doctors in Ireland.* BMJ Open, 2017. **7**(10).
16. Smith, S.E., et al., *Foundation Year 2 doctors' reasons for leaving UK medicine: an in-depth analysis of decision-making using semistructured interviews.* BMJ Open, 2018. **8**(3).
17. Hoffman, R.I. and A.D. Bonney, *Junior doctors, burnout and wellbeing Understanding the experience of burnout in general practice registrars and hospital equivalents.* 2018.
18. Casey, D. and K.A. Choong, *Suicide whilst under GMC's fitness to practise investigation: Were those deaths preventable?* Journal of Forensic and Legal Medicine, 2016. **37**: p. 22-27.
19. Laarman, B.S., et al., *How do doctors in the Netherlands perceive the impact of disciplinary procedures and disclosure of disciplinary measures on their professional practice, health and career opportunities? A questionnaire among medical doctors who received a disciplinary measure.* BMJ Open, 2019. **9**(3): p. e023576.
20. Bourne, T., et al., *The impact of complaints procedures on the welfare, health and clinical practise of 7926 doctors in the UK: a cross-sectional survey.* BMJ Open, 2015. **5**(1): p. e006687.
21. Nash, L., et al., *Psychological morbidity in Australian doctors who have and have not experienced a medico-legal matter: cross-sectional survey.* Aust N Z J Psychiatry, 2007. **41**(11): p. 917-25.
22. NHS., *Junior doctor morale. Understanding best practice working environments*. 2017, Health Education England.
23. Petrie, K., et al., *Working hours, common mental disorder and suicidal ideation among junior doctors in Australia: a cross-sectional survey.* BMJ open, 2020. **10**(1).
24. West, C.P., et al., *Association of resident fatigue and distress with perceived medical errors.* Jama, 2009. **302**(12): p. 1294-1300.
25. BMA, *BMA Fatigue and Facilities Charter.* 2018.
26. Gunasingam, N., et al., *Reducing stress and burnout in junior doctors: the impact of debriefing sessions.* Postgraduate Medical Journal, 2015. **91**(1074): p. 182-187.
27. Flanagan, E., et al., *Reflection for all healthcare staff: a national evaluation of Schwartz rounds.* Journal of Interprofessional Care, 2020. **34**(1): p. 140-142.
28. Bakker, A.B. and E. Demerouti, *Job demands-resources theory: Taking stock and looking forward.* J Occup Health Psychol, 2017. **22**(3): p. 273-285.
29. van der Doef, M. and S. Maes, *The Job Demand-Control (-Support) model and psychological well-being: A review of 20 years of empirical research.* Work & Stress, 1999. **13**(2): p. 87-114.
30. Spiers, J., et al., *Barriers, facilitators, and survival strategies for GPs seeking treatment for distress: a qualitative study.* Br J Gen Pract, 2017. **67**(663): p. e700-e708.
31. Kay, M., et al., *Doctors as patients: a systematic review of doctors' health access and the barriers they experience.* British Journal of General Practice, 2008. **58**(552): p. 501-508.
32. Shanafelt, T.D., et al. *Healing the professional culture of medicine*. in *Mayo Clinic Proceedings*. 2019. Elsevier.
33. Schein, E.H., *The corporate culture survival guide*. Vol. 158. 2009: John Wiley & Sons.

For peer review only

34. Charmaz, K., *Constructionism and the grounded theory method.* Handbook of constructionist research, 2008. **1**: p. 397-412.
35. PHP, *https://mysupportt.com/nhs-practitioner-health-programme/.* 2020.
36. Saunders, B., et al., *Saturation in qualitative research: exploring its conceptualization and operationalization.* Quality & quantity, 2018. **52**(4): p. 1893-1907.
37. Braun, V. and V. Clarke, *Using thematic analysis in psychology.* Qualitative research in psychology., 2006. **3**(2): p. 77-101.
38. Charmaz, K., *Constructing grounded theory. A practical guide through qualitative analysis*. 2006, London: Sage.
39. Patton, M., *Enhancing the Quality and Credibility of Qualitative Analysis.* HSR, 1999. **34**(5).
40. Tong, A., P. Sainsbury, and J. Craig, *Consolidated criteria for reporting qualitative research (COREQ): a 32-item checklist for interviews and focus groups.* International journal for quality in health care, 2007. **19**(6): p. 349-357.
41. Gottlieb, M., et al., *Impostor syndrome among physicians and physicians in training: A scoping review.* Med Educ, 2020. **54**(2): p. 116-124.
42. Garelick, A.I., *Doctors' health: stigma and the professional discomfort in seeking help.* The Psychiatrist, 2012. **36**(3): p. 81-84.
43. Henderson, M., et al., *Shame! Self-stigmatisation as an obstacle to sick doctors returning to work: a qualitative study.* BMJ Open, 2012. **2**(5).
44. van Pelt, F., *Peer support: healthcare professionals supporting each other after adverse medical events.* Quality and Safety in Health Care, 2008. **17**(4): p. 249.
45. Bruce, S.M., H.M. Conaglen, and J.V. Conaglen, *Burnout in physicians: a case for peer-support.* Intern Med J, 2005. **35**(5): p. 272-8.
46. Shapiro, J. and P. Galowitz, *Peer Support for Clinicians: A Programmatic Approach.* Acad Med, 2016. **91**(9): p. 1200-4.
47. Borrill, C., et al., *Team working and effectiveness in health care.* British Journal of Healthcare Management, 2000. **6**(8): p. 364-371.
48. West, M.A. and L. Markiewicz, *Effective team working in health care.* The Oxford handbook of health care management, 2016: p. 231-254.
49. GOV.UK., *NHS staff management and health service quality.* 2019.
50. *MIND, Introduction to mentally healthy workplaces* 2020.
51. Samsudin, E.Z., et al., *Organisational antecedents of workplace victimisation: The role of organisational climate, culture, leadership, support, and justice in predicting junior doctors' exposure to bullying at work.* The International Journal of Health Planning and Management, 2020. **35**(1): p. 346-367.
52. King's Fund, *NHS leadership and culture: our position.* 2020.
53. West, M. and D. Coia. *Caring for doctors Caring for patients*. 2019.
54. HEE, *Supporting Educators and Learners: Handbook for Educational Supervisors*. 2017.
55. Lanius, R.A., E. Vermetten, and C. Pain, *The impact of early life trauma on health and disease: The hidden epidemic*. 2010: Cambridge, UK.
56. Crumpei, I. and I. Dafinoiu, *The relation of clinical empathy to secondary traumatic stress.* Procedia-Social and Behavioral Sciences, 2012. **33**: p. 438-442.
57. Leigh-Hunt, N., et al., *An overview of systematic reviews on the public health consequences of social isolation and loneliness.* Public Health, 2017. **152**: p. 157-171.
58. Carrieri, D., et al., *Optimising strategies to address mental ill-health in doctors and medical students: 'Care Under Pressure' realist review and implementation guidance.* BMC Medicine, 2020. **18**(1): p. 76.
59. Steffens, N.K., et al., *A Meta-Analytic Review of Social Identification and Health in Organizational Contexts.* Personality and Social Psychology Review, 2016. **21**(4): p. 303-335.
60. Cook, J. and T. Wall, *New work attitude measures of trust, organizational commitment and personal need non-fulfilment.* Journal of Occupational Psychology, 1980. **53**(1): p. 39-52.

For peer review only

61. Leiter, M.P., A. Day, and L. Price, *Attachment styles at work: Measurement, collegial relationships, and burnout.* Burnout Research, 2015. **2**(1): p. 25-35.
62. Seidler, Z.E., et al., *The role of masculinity in men's help-seeking for depression: A systematic review.* Clinical Psychology Review, 2016. **49**: p. 106-118.

For peer review only

Consolidated criteria for reporting qualitative studies (COREQ): 32-item checklist

Developed from:
Tong A, Sainsbury P, Craig J. Consolidated criteria for reporting qualitative research (COREQ): a 32-item checklist for interviews and focus groups. *International Journal for Quality in Health Care*. 2007. Volume 19, Number 6: pp. 349 – 357

No. Item	Guide questions/description	Reported on Page #
**Domain 1: Research team and reflexivity**		
Personal Characteristics		
1. Interviewer/facilitator	Which author/s conducted the interview or focus group?	Page 7
2. Credentials	What were the researcher's credentials? E.g. PhD, MD	Page 7
3. Occupation	What was their occupation at the time of the study?	Page 7
4. Gender	Was the researcher male or female?	Page 7
5. Experience and training	What experience or training did the researcher have?	Page 7
Relationship with participants		
6. Relationship established	Was a relationship established prior to study commencement?	Page 7 .
7. Participant knowledge of the interviewer	What did the participants know about the researcher? e.g. personal goals, reasons for doing the research	Page 7
8. Interviewer characteristics	What characteristics were reported about the inter viewer/facilitator? e.g. Bias, assumptions, reasons and interests in the research topic	Page 7

For peer review only

**Domain 2: study design**		
Theoretical framework		
9. Methodological orientation and Theory	What methodological orientation was stated to underpin the study? e.g. grounded theory, discourse analysis, ethnography, phenomenology, content analysis	Page 7
Participant selection		
10. Sampling	How were participants selected? e.g. purposive, convenience, consecutive, snowball	Page 7
11. Method of approach	How were participants approached? e.g. face-to-face, telephone, mail, email	Page 7
12. Sample size	How many participants were in the study?	Page 9
13. Non-participation	How many people refused to participate or dropped out? Reasons?	None
Setting		
14. Setting of data collection	Where was the data collected? e.g. home, clinic, workplace	Page 8 .
15. Presence of non-participants	Was anyone else present besides the participants and researchers?	No
16. Description of sample	What are the important characteristics of the sample? e.g. demographic data, date	Page 9/19-20
Data collection		
17. Interview guide	Were questions, prompts, guides provided by the authors? Was it pilot tested?	Page 7/20
18. Repeat interviews	Were repeat interviews carried out? If yes, how many?	No, due to lack of resources
19. Audio/visual recording	Did the research use audio or visual recording to collect the data?	Page 7

For peer review only

20. Field notes	Were field notes made during and/or after the interview or focus group?	Yes, Page 8
21. Duration	What was the duration of the inter views or focus group?	Page 8
22. Data saturation	Was data saturation discussed?	Yes, page 8
23. Transcripts returned	Were transcripts returned to participants for comment and/or correction?	No, due to lack of resources
**Domain 3: analysis and findings**		
Data analysis		
24. Number of data coders	How many data coders coded the data?	Page 8
25. Description of the coding tree	Did authors provide a description of the coding tree?	No
26. Derivation of themes	Derived from the data?	Page 8
27. Software	What software, if applicable, was used to manage the data?	Page 8
28. Participant checking	Did participants provide feedback on the findings?	No, due to lack of resources
Reporting		
29. Quotations presented	Were participant quotations presented to illustrate the themes/findings? Was each quotation identified? e.g. participant number	Pages 8-15
30. Data and findings consistent	Was there consistency between the data presented and the findings?	Yes
31. Clarity of major themes	Were major themes clearly presented in the findings?	Yes
32. Clarity of minor themes	Is there a description of diverse cases or discussion of minor themes?	Yes

For peer review only

BMJ Open

Protective factors and sources of support in the workplace as experienced by UK foundation and junior doctors: a qualitative study

Journal:	*BMJ Open*
Manuscript ID	bmjopen-2020-045588.R2
Article Type:	Original research
Date Submitted by the Author:	16-Mar-2021

Complete List of Authors:	Riley, Ruth; University of Birmingham College of Medical and Dental Sciences<br>Kokab, Farina ; University of Birmingham,<br>Buszewicz, Marta; University College London, Research Department of Primary Care and Population Health<br>Gopfert, Anya; Oxford University Hospitals NHS Trust<br>Van Hove, Maria; London School of Hygiene & Tropical Medicine<br>Taylor, Anna<br>Teoh, Kevin; Birkbeck University of London, Department of Organizational Psychology<br>Martin, James; University of Birmingham, Institute of Applied Health Research<br>Appleby, Louis; University of Manchester, Psychiatry & Behavioral Sciences<br>Chew-Graham, Carolyn; University of Keele

**Primary Subject Heading**:	Mental health
Secondary Subject Heading:	Qualitative research, Occupational and environmental medicine, Medical education and training
Keywords:	QUALITATIVE RESEARCH, OCCUPATIONAL & INDUSTRIAL MEDICINE, MENTAL HEALTH, MEDICAL EDUCATION & TRAINING, Suicide & self-harm < PSYCHIATRY

SCHOLARONE™
Manuscripts

I, the Submitting Author has the right to grant and does grant on behalf of all authors of the Work (as defined in the below author licence), an exclusive licence and/or a non-exclusive licence for contributions from authors who are: i) UK Crown employees; ii) where BMJ has agreed a CC-BY licence shall apply, and/or iii) in accordance with the terms applicable for US Federal Government officers or employees acting as part of their official duties; on a worldwide, perpetual, irrevocable, royalty-free basis to BMJ Publishing Group Ltd ("BMJ") its licensees and where the relevant Journal is co-owned by BMJ to the co-owners of the Journal, to publish the Work in this journal and any other BMJ products and to exploit all rights, as set out in our licence.

The Submitting Author accepts and understands that any supply made under these terms is made by BMJ to the Submitting Author unless you are acting as an employee on behalf of your employer or a postgraduate student of an affiliated institution which is paying any applicable article publishing charge ("APC") for Open Access articles. Where the Submitting Author wishes to make the Work available on an Open Access basis (and intends to pay the relevant APC), the terms of reuse of such Open Access shall be governed by a Creative Commons licence – details of these licences and which Creative Commons licence will apply to this Work are set out in our licence referred to above.

Other than as permitted in any relevant BMJ Author's Self Archiving Policies, I confirm this Work has not been accepted for publication elsewhere, is not being considered for publication elsewhere and does not duplicate material already published. I confirm all authors consent to publication of this Work and authorise the granting of this licence.

For peer review only

Protective factors and sources of support in the workplace as experienced by UK foundation and junior doctors: a qualitative study

Ruth Riley < Corresponding author>

Institute of Applied Health Research

University of Birmingham
Birmingham

United Kingdom

r.riley@bham.ac.uk

Tel: + 44 (0) 121 414 8549

Farina Kokab,

Department of Social Work and Social Policy

School of Social Policy

University of Birmingham

Birmingham

United Kingdom

Marta Buszewicz,

Research Department of Primary Care and Population Health

University College London

London

United Kingdom

For peer review only

Anya Gopfert

Oxford University Hospitals Trust

Oxford

United Kingdom

Maria van Hove

London School of Hygiene and Tropical Medicine

London

United Kingdom

Anna Kathryn Taylor

Leeds Institute of Health Sciences

Faculty of Medicine and Health

University of Leeds

Leeds

United Kingdom

Kevin Rui-Han Teoh,

Department of Organizational Psychology
Birkbeck, University of London

London

United Kingdom

For peer review only

For peer review only - http://bmjopen.bmj.com/site/about/guidelines.xhtml

James Martin

Institute of Applied Health Research

University of Birmingham
Birmingham

United Kingdom

Louis Appleby

Division of Psychology and Mental Health

School of Medicine

University of Manchester

Manchester

United Kingdom

Carolyn A. Chew-Graham

School of Medicine

Keele University

Keele

United Kingdom

For peer review only

For peer review only - http://bmjopen.bmj.com/site/about/guidelines.xhtml

Abstract

Objectives. This paper reports findings identifying foundation and junior doctors' experiences of occupational and psychological protective factors in the workplace and sources of effective support.

Design. Interpretative, inductive, qualitative study involving in-depth interviews with 21 junior doctor participants. The interviews were audio-recorded, transcribed, anonymised and imported into NVivo11 to facilitate data management. Data were analysed using a thematic analysis employing the constant comparative method.

Setting. National Health Service in the United Kingdom.

Participants. Participants were recruited from junior doctors through social media (for example, the BMA junior doctors' Facebook group, Twitter and the mental health research charity websites). A purposive sample of 16 female and 5 male, ethnically diverse, from a range of specialities, across the UK. Junior doctor participants self-identified as having stress, distress, anxiety, depression and suicidal thoughts or having attempted to kill themselves.

Results. Analysis identified three main themes, with corresponding subthemes relating to protective work factors and facilitators of support: 1. Support from work colleagues – help with managing workloads and emotional support. 2. Supportive leadership strategies, including feeling valued and accepted, trust and communication, supportive learning environments, challenging stigma and normalising vulnerability 3. Access to professional support – counselling, CBT and medication through General Practitioners, specialist support services for doctors, and private therapy.

Conclusions. Findings show that supportive leadership, effective management practices, peer support, and access to appropriate professional support can help mitigate the negative impact of working conditions and cultures experienced by junior doctors. Feeling connected, supported and valued by colleagues and consultants acts as an important buffer against emotional distress despite working under challenging working conditions.

Keywords: Qualitative, Hospital Staff, Occupational Stress, Mental Health, Suicide, Psychological Support

Word count: 3889

For peer review only - http://bmjopen.bmj.com/site/about/guidelines.xhtml

Strengths and limitations of this study

- Few qualitative studies have explored work-related psychological distress in junior doctors; we therefore employed in-depth interviews with 21 junior doctors from across the United Kingdom to explore this distress

- The sample included five male participants compared with 16 female participants which may be a limitation in this study; however, the purposive sampling, of participants, ensured the sample was varied in terms of ethnicity, number of years in training, speciality, and geographical location. .

- Participants self-identified as having chronic stress and/or mental health problems, including anxiety and depression; therefore the findings may not be generalizable to the general population of junior doctors.

- Semi-structured interviews generated rich qualitative data and an iterative process of concurrent data collection and constant comparative analysis facilitated the simultaneous exploration, refinement and enrichment of key themes.

INTRODUCTION

There are currently 115,376 doctors working in the NHS, almost half of whom (56,404) are termed 'junior doctors', which includes doctors in specialist training or at pre-Consultant grade, and Foundation Year doctors [1]. Previous research indicates that doctors are vulnerable to chronic stress, anxiety, depression and burnout [2, 3]. Severe depression has also been identified as a risk factor for suicide [4, 5]. High rates of suicide have frequently been reported among doctors, but with considerable heterogeneity over time and between countries [6]. Despite mixed findings about suicide rates internationally, there are concerns over suicidal ideation and behaviour in doctors, particularly women [7, 8]. International research indicates that psychological distress (such as depression or anxiety) is higher in female compared to male doctors [9].

The Practitioner Health Programme (PHP) in London, an England-wide specialist mental health, drug and alcohol service for doctors and dentists, reports that two-thirds of those presenting are women, with higher attendance rates among young female doctors [10]. It is uncertain whether this is representative of the general population of doctors or reflects gender differences in help-seeking behaviour.

Most previous research into distress and suicide in junior doctors reports quantitative, retrospective studies, with the focus on the individual risk factors (particularly emphasising the need for 'resilience'), [11, 12] and overlooking the wider systemic, organisational and

For peer review only - http://bmjopen.bmj.com/site/about/guidelines.xhtml

cultural sources of distress among this population of health professionals. Focusing on individual risk factors and interventions are likely to detract from considering solutions or interventions targeting the learning environment, organisational culture and systemic factors [13, 14]. To our knowledge, no previous study has qualitatively examined how junior doctors view their working conditions (e.g. rotas, spaces to rest, eat and sleep when on-call) and work cultures (relationships, support, job control), and the factors (e.g. debriefs following critical incidents, policies, available support, management practices) which may protect them from psychological distress or offer them support.

Junior doctors face specific pressures related to their professional stage and development. In a nationwide survey of junior doctors in Ireland, stress, anxiety and depression were highest among junior doctors as compared to consultants [15]. A study exploring junior doctors' reasons for leaving medicine in the UK cited lack of support, mentorship or formal training, loneliness and bullying [16]. A qualitative study undertaken in Australia found that lack of support from colleagues, working beyond one's perceived abilities and neglecting self-care were key contributors to burnout and reduced wellbeing among junior doctors [17]. Doctors under investigation are also at higher risk of suicide and psychological distress [18, 19]; those in receipt or facing threats of complaints experience elevated anxiety, depression and suicidal thoughts compared to colleagues who face no complaints [20, 21].

A lack of support, mental health stigma, and toxic work cultures, characterised by bullying and a blame and shame culture were identified as a key source of distress among junior doctors, in a separate paper linked to this study [Riley et al. 2020 – under review BMJ Open]. Similarly, feeling undervalued, unsupported or having reduced autonomy were key factors affecting low morale among junior doctors [22]; this highlights the impact of working conditions and work culture on junior doctors and the potential value of providing effective support and supervision.

There is substantial evidence linking longer working hours with mental ill health and reduced patient safety; junior doctors who worked over 55 hours a week were more than twice as likely to report a common mental health problem [23] and increased likelihood of making a medical error [24]. This highlights the importance of providing suitable facilities for sleep, rest and social spaces, and to facilitate social interaction and access to collegial support [25].

Evidence has found that junior doctors valued the emotional support derived from attending debriefing sessions following adverse events, although its effectiveness in reducing burnout was equivocal [26]. Workplace interventions such as Schwartz rounds, which provide permission and a safe space for healthcare staff to talk about the range of feelings and challenges which arise during their work while also fostering connectedness to others, were valued by staff [27]. Although numerous workplace health theories [28, 29] identify effective support as a core antecedent to positive mental health and as a potential buffer against challenging work environments, how this might manifest amongst junior doctors is unclear.

For peer review only - http://bmjopen.bmj.com/site/about/guidelines.xhtml

Identifying such patterns or contextual factors is crucial in developing targeted improvements or interventions that can reduce the risk of mental ill health and psychological distress from the outset.

Occupational or work-related stress are also compounded by barriers to help-seeking which include difficulties accessing healthcare due to working hours, perceived stigma, concerns about loss of confidentiality, therapeutic nihilism (a cynicism about the effectiveness and availability of existing interventions for mental ill health) and pressure to continue working while unwell, due to staff shortages and concerns about letting colleagues down [2, 9, 30, 31].

Facilitators included the availability of confidential, specialised services for doctors, emotional and practical support such as finding cover for shifts [30]. Shanafelt et al. [32] recommend various interventions (e.g. positive role models, training of group and teams, inter-professional support groups) to facilitate culture change. Promoting wellbeing by providing psychological safety and positive learning environments may reduce learning anxiety among junior doctors [33].

This qualitative study is part of a wider mixed-methods study examining the psychological, cultural and occupational contexts associated with reduced psychological wellbeing in junior doctors, and protective factors which may mitigate against this. This paper reports the qualitative findings focussed on the protective factors and sources of effective support in the workplace as experienced by junior doctors.

METHODS

Study design and setting

The study methodology was underpinned by a constructivist grounded theory approach [34], using qualitative methods, with semi-structured interviews to explore junior doctors' perspectives and experiences of stress and distress. The study setting was the NHS in the UK.

Sampling and Recruitment

We used a range of recruitment methods including advertisements on social media (for example, the junior doctors' Facebook group, Twitter and the mental health research charity websites). In addition, information about the study was circulated through the Practitioner Health Programme, a specialised mental health and drug and alcohol service dedicated to doctors and dentists [35].

Potential participants were asked to express their interest by contacting members of the study team who then provided further information about the study.

The research team employed purposive sampling from the responding junior doctors, to ensure maximum variation taking account of the following characteristics: gender, age,

For peer review only - http://bmjopen.bmj.com/site/about/guidelines.xhtml

For peer review only

ethnicity, sexuality, geographical location, different grades/duration of General Medical Council registration, medical speciality, disclosure of a mental health diagnosis, individuals who reported self-harm behaviour, had thoughts of suicide or had attempted to kill themselves.

The eligibility criteria used are listed in Box 1.

Junior doctors interested in taking part were sent a reply slip and consent form electronically and asked to sign the consent form prior to the interview, before returning it to the study researcher. Face-to-face, telephone or 'virtual platform' interviews were arranged at a time convenient to each participant. They were given the opportunity to raise questions prior to being interviewed and during the interview.

At the end of each interview, the researcher informed the participant about the next steps, how their data would be used, and checked on their wellbeing prior to leaving the interview setting. A risk protocol was used to ensure appropriate support was provided to participants (and the researcher) in the event of the disclosure of significant distress or suicidal ideation.

Data collection

A topic guide (see box 2) was developed by the research team to generate discussion in the semi-structured interviews. The topic guide was informed by the existing literature, input from junior doctors on the study team, and patient and public involvement (PPI) input (see below), and modified iteratively as data collection and analysis progressed. The topic guide aimed to capture participants' views, experiences, feelings and beliefs about working conditions and cultures which were perceived to be stressful or distressing, and factors which were perceived/felt to be protective.

One-to-one interviews were conducted face-to-face (n=7), by telephone (n=13) or using a digital virtual platform (n=1) and digitally recorded with consent. The in-depth interviews were conducted by two researchers (FK, RR), both female social and behavioural scientists with qualitative methods expertise. The recorded interviews were transcribed verbatim and checked for accuracy of transcription by the study researcher before analysis. All transcripts were anonymised before discussion within the wider research team. Reflexive notes were recorded throughout the process. Recruitment and data collection was continued until data saturation was judged to have been achieved [36].

For peer review only - http://bmjopen.bmj.com/site/about/guidelines.xhtml

Data analysis

Analysis began with data collection, and was iterative and inductive, employing the constant comparative method [37, 38] until theoretical data saturation was achieved, such that no new analytic categories emerged [36].

The research team included three junior doctors (AG, AKT, MvH), two academic general practitioners (CCG, MB), one occupational health psychologist (KT) and two social scientists (RR and FK). FK coded the data set; RR coded a sub-sample and contributed to the organisation of themes. The multidisciplinary team provided commentary on transcripts to generate and refine codes and thematic categories and provide researcher triangulation, thereby increasing the credibility of the research findings [39]. Data were managed using NVivo 11. The study is reported in line with the consolidated criteria for reporting qualitative research (COREQ) [40].

Patient and Public Involvement

There were three junior doctors on the research team, all of whom consulted with colleagues about the initial research idea. It was felt important that the research focus needed to explore working conditions and cultures, rather than focusing the gaze inward or on individuals. Five junior doctors provided feedback on the initial funding application, and four of these gave feedback on the protocol, topic guide and participant facing documents. Junior doctors have been actively involved in publicising the study to junior doctors and we are working on a dissemination strategy with junior doctors outside the research team. Due to the time constraints of junior doctors, PPIE members were consulted via email and telephone.

FINDINGS

Twenty-one interviews were conducted, lasting between 43 and 103 min (mean= 65 min), between November 2019 and May 2020. The demographic and professional characteristics of participants are included in Table 1. Analysis of the interview transcripts and field notes identified three main themes, with corresponding subthemes relating to protective work factors and facilitators of support: 1. Support from work colleagues, including help with managing workloads and emotional support. 2. Supportive leadership strategies, including feeling valued and accepted, trust and communication, supportive learning environments and challenging stigma and normalising vulnerability 3. Access to professional support, including Cognitive Behavioural Therapy (CBT), counselling and medication through General Practitioner and specialist support services for doctors, and accessing private counselling/therapy.

SUPPORT FROM WORK COLLEAGUES

Many participants emphasised the importance of working in protective and supportive work cultures, characterised by good interpersonal relationships, a strong team morale where

For peer review only - http://bmjopen.bmj.com/site/about/guidelines.xhtml

there was a shared responsibility for workloads, and where staff felt supported with the clinical components of their work and the emotional impact of their job. Crucially, participants highlighted the interdependence between collegial support, supportive work cultures and their emotional wellbeing and ability to cope with the demands of their job.

a) Help with managing workloads

Participants identified the benefits of having mutually supportive interpersonal connections at work where there was a willingness to share responsibilities, offer practical support, helping with workloads or specific tasks:

"I've made good relationships… [name of speciality] was actually having a very, very nice group of colleagues on the whole and supporting each other. Like offering help if we're struggling, if someone's struggling with a task then I would offer help and vice versa." (JD12, male)

The following participant highlighted that nurses offered support by undertaking tasks which free up time for junior doctors to focus on clinical work: Spending time with supportive staff could alleviate some of the burden and stress that junior doctors may be experiencing, especially in new and unfamiliar roles.

"My relationships with my colleagues, both nurses and doctors, are incredible. I couldn't say a bad word about any of them. They have been so wonderfully supportive. The nursing staff have carried me in every job I've been on. They've carried me because they're the constant and they're there all the time." (JD17, female)

b) Emotional support

Participants iterated the importance of peer support and feeling emotionally supported within the team, for example, in response to critical incidents, or being bullied by a colleague::

"A couple of weeks ago my friend was having a terrible time with a registrar who was being hideous to her and so, you know we kind of helped support her through that and empathise with her and you know I think that's really helpful." (JD20, female)

The following participant referred to the '*in it together*' team spirit and camaraderie which acted as an emotional buffer when working under challenging working conditions:

"We were all in it together and actually, the working culture is really, really good … even in the rubbish conditions that we're working in, it's the people that have made it worthwhile and they've helped me cope with things, so if I'm having a bad day or I'm a bit down, actually it's the camaraderie of working with midwives, other doctors, nurses, pharmacists, porters that have made it quite a happy thing to do really." (JD09. female)

Many participants had contrasting experiences on different rotations; the following participant described her experience of being in a supportive work culture in the context of a

For peer review only - http://bmjopen.bmj.com/site/about/guidelines.xhtml

critical incident, which she compared with the ‘blame culture’ experienced on a previous rotation:

“In the unit I'm in at the moment, it's a lot more about learning rather than blaming. I think you feel safer then to express opinions or to discuss cases. You want to find out who was involved in order to be able to support them and that’s the feeling that you get from the senior midwives and the consultants you work with… The unit that I was in before was really challenging in terms of feeling like you wouldn't be supported, even if you'd done nothing wrong.” (JD13, female)

Another participant reported feeling ‘*emotionally healthy*’ which she attributed to the supportive work culture. She contrasted this with a previous rotation where the cultural norm was to disengage with challenging emotions, leaving staff with unprocessed trauma:

*“I feel really emotionally healthy now. Cause I'm in such a supportive culture. But I look now particularly at [name of specialty] culture as a lot of people who, with a lot of unprocessed trauma. Huge amounts of unprocessed trauma. Amazing really that whole profession can be so in denial of the emotional impact of their job.” (*JD23, female)

The value of working in a supportive work culture was further highlighted by the following participant who recollected that having greater support from colleagues in her previous post would have reduced her sense of anxiety:

“I think if I’d have had that [informal support from colleagues] support in first year of someone who could listen, maybe I wouldn’t have been so anxious about everything.” (JD09, female)

The following participant suggested that, despite the potential stigma associated with mental illness, peer-support was often the first line of support for colleagues:

“I do think peer support is very important and I think that’s probably the first line for a lot of people, like even with some stigma and embarrassment about mental health, I think most people would usually turn to a colleague first.” (JD16, female)

SUPPORTIVE LEADERSHIP STRATEGIES

a) Feeling valued and accepted

This reoccurring theme refers to supportive and proactive leadership and management approaches to providing support to the team and promoting positive mental health. Participants relayed the importance of feeling valued, included and accepted as part of the team by the consultants. This included receiving recognition and praise for their efforts and

For peer review only - http://bmjopen.bmj.com/site/about/guidelines.xhtml

contributions, particularly when working in challenging, highly pressurised environments and working long shifts:

'… it [first job] was over 12 hours every single day, you know I think I started to cry because just at least someone was acknowledging the fact that we were doing 12, 13 hour days every day … it was extremely hard but…there was a very good group of consultants who just acknowledged that it was hard, there was not much they could do about it, they had to work hard as well but they were very, very, very supportive of that… they [consultants] just knew what your name was, they knew your role, you felt valued there." (JD01, male)

"…it makes such a difference and then also like being empowered to be of good patient care, so you know not being shouted at if you've taken half an hour to see a patient and actually you know being recognised, 'Oh you did a really thorough job actually, well done, good for you' and kind of you know just actually some recognition of what you're doing. Yeah, that's what is good, I guess." (JD20, female)

This participant emphasised the importance of feeling valued by consultants or patients and its relationship to job satisfaction and confidence:

"…when you receive some good feedback, you can really get a bit of a buzz and a bit of a high from that, either from a patient or from a colleague when they said, you know, 'you've really made a difference today' or 'you've really helped me today' or 'you've made me feel better, thank you'. Whenever you get something like that, which doesn't happen all the time by any means, but when you get a bit of feedback, it makes you realise why you're doing it and I guess all those feelings of anxiety and the feeling of not being good enough, which is a big thing for me. So, if somebody's appreciative, I think that can brighten your day really and make you feel like you're worth something." (JD09, female)

b) Trust and communication

A few participants recalled the value of consultants who were proactive in their support of staff, openly inviting them to discuss concerns, ask questions or to enquire about their work and wellbeing. Establishing these open channels of communication and being approachable made it easier for participants to seek support or advice when needed:

"Whereas, the two consultants that I go to for that sort of support always made it clear that they were open to talking about anything that was bothering me. They continued to keep that relationship open, even when I wasn't working with them anymore. It was just the odd touch base email like, 'How are things going? What are you up to? Let's have a coffee one day'. It was that kind of thing. You're made to feel a bit less like you're onerous or that you're bothering them when you ask them about something." (JD13, female)

Such was the importance of supportive relationship with consultants, a few participants had maintained those connections after moving to a new post:

For peer review only - http://bmjopen.bmj.com/site/about/guidelines.xhtml

For peer review only

"I've got a couple of people that I go back to when I need support ….and I know will speak to them both but I will always go back to those few people that I trust and I know." (JD19, female)

c) Supportive learning environments

Participants reflected on work cultures where there was a shared commitment to team learning. Such an ethos enabled people to practice without fear of reprisal, to reflect on their work, and to view errors as opportunities for learning and improvement:

"*My ST1 [*Specialist Training year one*] year was extremely enjoyable, because again it was a very supportive place to work and they encouraged you to ask questions and didn't criticise and there was a real sort of no blame culture*" (JD01, male)

Some participants suggested that cohesiveness within teams could be enabled through multidisciplinary training opportunities. These events facilitated a shared understanding of different roles within the team and awareness of the challenges and concerns faced by different professional groups:

"Where I am at the moment is really good. The current unit I'm in has a lot of multi-professional training that isn't necessarily clinical. We have multi-professional leadership days where the senior junior doctors at ST5 level plus and the senior midwives all get together to do the training together. I think that really helps because we understood their role and their concerns when they're coordinating a unit and they understand that we can't really do everything" (JD13, female)

d) Challenging stigma and normalising vulnerability

A number of participants emphasised the role of consultants and supervisors in de-stigmatising mental ill health by sharing or disclosing their vulnerability or experiences of mental ill health to their staff; participants report that this created a more open and authentic work culture where staff also felt safe to disclose their vulnerability:

"We were working a Sunday together and I said, 'I haven't seen you in a while.' She said, 'No, I have been off for about four months.' I said, 'Are you just coming back?' She said, 'Yeah, I was off because I had a mental breakdown. One day, I just realised that I was really, really unwell and I just had to go home and I didn't return for a number of months until I got myself sorted out.' My point was just her talking about that, I think, does wonders because she's a woman who has been there forever. She's a senior consultant. Just telling other people, 'Yeah, I've gone through the struggles. I need medication for this, that and the other. It's okay. I'm still here functioning and it's fine'. I think people need to be more open about that," (JD03, male)

For peer review only - http://bmjopen.bmj.com/site/about/guidelines.xhtml

"I think what really needs to change is that I think that more senior doctors need to be open about their struggles and their issues in training because I think there's a perception that you need to be invulnerable among more junior trainees in particular, which I think as people gradually have breakdowns, they're robbed of that illusion. Yes, yes, I think that kind of cultural change needs to kind of happen." (JD07, female)

ACCESS TO PROFESSIONAL SUPPORT

Few participants reported seeking formalised help from within the workplace (e.g. occupational health) but some study participants had sought professional help from counsellors, therapists, general practitioners (GPs) and specialist services for doctors, outside the workplace. The following participant was prescribed antidepressants by her GP to manage her distress:

"…I was in tears and nervous …I kept on saying, 'I don't feel like this is going to ever change. I don't feel confident,' and then I started antidepressants and incredibly, within about two weeks, I was sleeping. I felt better and I felt calm. I realised just how sick I had been." (JD08, female)

Accessing external support was not without barriers. The following participant highlights the significant delay in accessing a counselling referral through their GP:

"My GP referred me for local support and it took them a year to contact me…I mean, it's just the NHS, that's how it is…and even then, it would be one o'clock, two o'clock, it would be nigh on impossible to actually access," (JD19, female)

Having access to services tailored to the needs of junior doctors was important for participants:

"Because you know that their [specialist service] *whole patient group are doctors and they've got such expertise of talking to doctors and professionals with the same issues, there is none of that having to make an excuse for yourself. We're so often held up in society as a really responsible, clever and respected group that you can be vulnerable in the PHP. That was really, really validating.*" (JD08, female)

The following participant found out about the Practitioner Health Programme (PHP) by chance (a family member found a PHP leaflet in a waiting room); she was concerned about being referred to a psychiatrist in her local trust because she was planning on undertaking a rotation there in the future:

"There is the Practitioner Health Programme… I've been seeing a GP, that lives down the road from me, who's absolutely amazing…I feel like that's a really good setup…I didn't feel

For peer review only - http://bmjopen.bmj.com/site/about/guidelines.xhtml

comfortable being treated in the mental health trust here and also, I want to look at working in that mental health trust. Being able to move my care completely away from that is very important. I didn't know that was there at the beginning. That's when I ran into some of my problems and when I wasn't seeking the help I needed because I didn't know that I could have anonymity." (JD22, female)

Due to concerns about the potential loss of confidentiality, the following participant self-referred to a private clinic to seek the formal support they needed:

"I went to a private mental health clinic and I referred myself, I phoned up and (? they) said well the Consultant probably would take a self-referral especially if you're a Doctor, so I wrote my own referral letter and I went privately to the Psychiatrist across the road from the hospital and he got me some treatment which was mainly in the form of counselling … And that changed everything, you know that turned everything around." (JD15, female)

A few participants felt it would be beneficial if junior doctors had access to monthly therapy or supervision sessions, either group work or one-to-one, a safe space to share and process the emotional impact of the job and seek support:

"If, as junior doctors, you saw a therapist once a month... if we made it universal (which is very easy to say) that for one hour a month, everyone had to see someone to talk to, I think that would be a good way of moving forward. If there was something that was really serious, it's a safe space and we can talk about it. If it needs to be escalated or if any sort of treatment needs to be started in whichever way that might be, go for it." (JD03, male)

"…sometimes lots of stuff coming up and yet there's absolutely nothing. For me I feel like, every doctor should have some form of emotional supervision." (JD23, female)

DISCUSSION

The findings highlight the value of support from work colleagues and work cultures engendered by camaraderie and connectedness with colleagues. The emotional support offered by colleagues often enabled junior doctors to manage the challenges of the job and was considered to be potentially protective against mental ill health. Participants felt more able to cope with the challenges of their work when their managers and leaders valued staff, offering clinical and emotional support and creating positive learning environments which reduce learning anxiety by providing psychological safety [32, 33]. Positive learning cultures, characterised by social support, validation of success, and positive affirmation have been found to be protective against burnout [41]. These findings also support the job-demand-resources theory [28]; namely, that key resources such as support from colleagues, quality of the relationships with supervisors/consultants and feeling valued can buffer the impact of

For peer review only - http://bmjopen.bmj.com/site/about/guidelines.xhtml

those demands related to their work (long hours, high workload) and consequently protect against burnout.

Our findings also highlight the role of consultants and other senior staff in de-stigmatising mental ill health by acknowledging and sharing their own vulnerability and creating a safe and authentic work culture which enabled staff to seek support. Promoting emotionally open cultures which facilitate disclosure by de-stigmatising mental ill health and allowing for vulnerability, empowers staff to seek support and could reduce the sense of isolation and shame often experienced by junior doctors [30]. Challenging and addressing stigma in medical school and beyond is a crucial stepping stone in enabling doctors to seek help without fear of reprisal or shame [9, 42, 43].

These findings highlight the positive impact of harnessing existing workforce capital (i.e. the skills and training of staff) to sustain cultures, practices and leadership styles which promote and engender supportive working environments and relationships. Participants in this study valued the support provided by work colleagues – this is underpinned by evidence attesting the value and importance of social relationships at work in buffering the demands of the job [44]. Belonging, feeling valued, and connectedness are protective factors and can promote positive mental health and a positive professional identity; for instance, peer/colleague support in the workplace has been found to mitigate the negative psychological consequences of adverse events [44, 45]. Authors recommend checking-in with colleagues, listening, reflecting, reframing, sense-making, coping, closing, and resources/referrals which can reduce the isolation and sense of shame experienced by colleagues [46].

Our findings highlight the value of working in supportive, cohesive and connected teams and are supported by previous research which found that staff who work in cohesive teams have better mental health, while lack of leadership was associated with poorer mental health [47, 48]. Having continuity in teams, including a named consultant fosters a sense of belonging and produces better patient outcomes [48, 49].

Participants in this study highlighted that, despite challenging working conditions - long hours, high workloads – they felt better able to cope with these demands and less isolated when they operated within a supportive work culture. Such cultures were often enabled and enacted by good leadership; consultants with proactive leadership and management styles which allow staff to feel supported by being valued, involved, heard and respected. Consultants who recognise and value the skills, knowledge and experience of team members, invite staff to ask questions and advertise their availability, can facilitate learning and empower staff to ask questions or for help, if needed. Such examples of effective management and leadership are supported by evidence which has shown that effective

For peer review only - http://bmjopen.bmj.com/site/about/guidelines.xhtml

leadership can promote mental health and wellbeing, prevent stress and enable staff to perform at their optimum [50].

Training for leadership styles and strategies which promote team cohesion or which, conversely, reduce the antecedents of toxic work cultures, including bullying, role conflict, role ambiguity, and chaotic and unpredictable work environments [51], is crucial. Employers also need to place greater value on meaningful, inclusive and impactful leadership and management styles which support staff [52]. However, NHS policy makers must also ensure that efforts are made to deliver sustainable workforce cultures which avoid an over-dependency on individual leaders. A whole systems approach to prevention and provision will need to be adopted to promote healthy workplaces for all NHS staff. This includes addressing systemic causes of stress such as workforce shortages, rota gaps, and improving physical working conditions, while also promoting work cultures which enable help-seeking and foster belonging and connectedness [13].

Working in effective and supportive teams requires staff to have shared objectives, values and continuity, necessitating planning and changes to rotas which can help to ensure junior doctors feel part of a team [53]. There is substantial evidence that working in teams which lack continuity in staff or shared objectives is detrimental to the mental health and wellbeing of staff and impacts negatively on patient care and safety [48, 49]. Junior doctors need to feel valued, capitalising on existing support infrastructure and assets (i.e. teams, supervisors, colleagues) to provide clinical and psychological support (e.g. interprofessional debriefs for processing and validating feelings). Organisations will need to provide the training and coaching to develop effective and cohesive teams, while also meeting recommendations set out in existing guidance to ensure the clinical, learning, training and emotional needs of doctors are met [54].

Findings from this study, reported in a sister paper [Riley et al.2020a in press], revealed the potential for emotional distress when junior doctors work in contrasting cultures where there are no safe spaces to discuss difficult or traumatic work experiences, such as the sudden or violent death of a patient. Feeling supported, permitting time out or time off work when needed, and minimising any potential for isolation and distress in such circumstances can reduce the likelihood of secondary/vicarious trauma (a process of change resulting from the effects of prolonged exposure to human suffering and trauma) [55, 56]. Feeling isolated when distressed is also a risk factor for suicide [57]; providing effective support needs to be an imperative in such contexts.

The provision and access to confidential support was important to participants and evidenced by previous research [2, 30]. Being able to find a safe, secure, anonymous and easy to access source of support outside of their work environment was greatly valued. To ensure doctors are aware of the available support, signposting information about specialist services such as

For peer review only - http://bmjopen.bmj.com/site/about/guidelines.xhtml

the Practitioner Health Programme could be included in every induction session across the country, and be reinforced by specialist training schools through Health Education England. Some participants however preferred accessing a trusted GP. Managers and leaders therefore need to facilitate and sanction appropriate support for staff when needed.

Participants' experiences in this study are supported by the findings of a realist review of interventions and strategies to reduce mental ill health and distress in doctors. This found that leadership and supervision level interventions, supported by the organisation, focusing on relationships, emotional support, and belonging were more likely to promote wellbeing and that fair and equitable learning environments which balanced positive/negative feedback enabled doctors to thrive [58]. This is underpinned by evidence which found that social connectedness contributes to the reduction of stress levels while social identification (belonging), is beneficial to the psychological health of employees and reduces burnout [44, 53, 58].

Leadership and management can play a key role in shaping group identities and belonging within a team [59]. Trust, defined as, "the extent to which one is willing to ascribe good intentions to, and have confidence in, the words and actions of other people" [60] has been shown to be positively associated with performance, civility and psychological safety [61]. Workforce cultures and environments play an integral role in promoting the mental health of junior doctors through support and creating safe and authentic environments where doctors can excel in their training, and overcome challenges linked to the demands of their job, enabling them to stay well.

This study has some limitations. There is a notable disparity in gender, with a higher proportion of female doctors participating. We received higher expressions of interest from female (n=37) participants compared to male (n=22), with a higher proportion of female participants then agreeing to participate in an interview. The interest in this study and increased willingness amongst female participants to come forward and talk about their experiences may reflect evidence indicating that female doctors are more likely to experience distress, with increased rates of suicide evidenced in young female doctors. The higher proportion of female participants may also reflect gendered help-seeking behaviour for mental ill health, evidenced also in the wider population [62]. Another potential limitation relates to the lower number of participants who reported having had suicidal thoughts (n=5) and participants who disclosed that they had made self-harm attempts (n=2).

CONCLUSION

Our findings show that support from colleagues, effective and supportive leadership and management practices and access to appropriate professional care can help mitigate the negative impact of working conditions and cultures experienced by many junior doctors.

For peer review only - http://bmjopen.bmj.com/site/about/guidelines.xhtml

Feeling connected, supported and valued by colleagues and consultants acts as an important buffer against emotional distress despite working under challenging working conditions and reduces any potential for isolation.

Authenticity of interactions between senior and junior colleagues was seen as an important aspect of how mental health and well-being are understood and negotiated in the work environment. Normalising vulnerability through disclosure and creating emotionally open cultures where vulnerability is accepted and understood, allows junior staff greater confidence to be open about factors affecting their own wellbeing and to seek and receive support when needed. Supporting doctors who request time out or time off and facilitating access to support could reduce the potential for isolation in the workplace and reduce stigma-related barriers to help-seeking. Examples of effective interventions and solutions to minimise distress and support staff are evidenced in existing leadership and collegial support, but need to be more consistently practiced across the NHS.

Author Contributions:

RR, CCG, MB, KT, AG, AKT, MvH, LA, JM – substantial contributions to conception and design

RR, FK, CCG, MB, KT, AG, AKT, LA, MvH, acquisition of data, or analysis and interpretation of data; drafting the article or revising it critically for important intellectual content; and final approval of the version to be published.

Acknowledgements: The authors would like to thank all volunteer junior doctor participants who kindly gave their time to share their experiences of working in in the NHS. We also wish to thank members of the PPIE group who provided valuable input throughout the study.

Competing interests: None

Funding: The study was funded by NIHR Research for Patient Benefit (PB-PG-0418-20023)

Department of Health Disclaimer: The views and opinions expressed therein are those of the authors and do not necessarily reflect those of the NIHR, NHS or the Department of Health.

Ethical approval: Ethical approval was granted by the University of Birmingham and Health Research Authority (reference number: 19/HRA/6579)

Data sharing statement: This study has not received ethical approval to share confidential data with any third party other than the study research team.

For peer review only - http://bmjopen.bmj.com/site/about/guidelines.xhtml

Tables/Boxes

Box 1: Inclusion and exclusion criteria

**INCLUSION CRITERIA**	**EXCLUSION CRITERIA**
Currently a foundation or junior (pre consultant) doctor working in a hospital in the NHS in England/Wales within the last 2 years – this covers any pre-consultant doctor working in the NHS. They do not necessarily have to be on a training contract.	Currently experiencing acute severe mental illness such as psychosis
Experience (within the last 4 years) experience of stress, distress, mental illness, self-harm (e.g. cutting, overdoses), suicidal thoughts, feelings, intent	Currently in receipt of drug and alcohol services (this does not include doctors who currently use alcohol/drugs to self-medicate such as doctors who may be using alcohol/drugs on a regular basis to help them relax but would not be classified as having an addiction problem.)
Has capacity to provide informed consent (this will be assumed given they are healthcare staff)	Actively suicidal or anyone who has made a suicide attempt within the last 6 weeks - to avoid causing any additional psychological harm during a period of acute distress/vulnerability

Table 1: Participant characteristics

**Participant characteristics**	**N=21**	**%**
Gender (female)	16	76
Age (years)		
20-29	10	48
30-39	11	52
Ethnicity		
Asian (other)	2	10
Bangladeshi	1	5

For peer review only - http://bmjopen.bmj.com/site/about/guidelines.xhtml

Chinese	1	5
Indian	3	14
White	13	62
White (other)	1	5
Sexual orientation (heterosexual)	15	
Years since qualification		
0-5	10	48
6-10	9	42
11-15	2	10
Specialty		
Emergency medicine	2	10
Medicine (including acute, diabetes/endocrinology, geriatrics)	9	42
Obstetrics and gynaecology	6	28
Paediatrics	2	10
Psychiatry	1	5
Public health	1	5

For peer review only

Box 2: Interview topic guide

Introduction and Background

- Describe general less stressful / more stressful jobs and the difference between these

Work Environment

- Describe main sources of stress in day-to-day working life
- Explore wider sources or stress not already mentioned
- Explore which jobs are more stressful

Impact of work on mental health and wellbeing

- Past, present and future outlook as to how work impacted / may impact mental health and wellbeing?

Preventing / Seeking / Managing help

- Explore management of workload / stress in day-to-day work life
- Discuss help seeking for distressing events
- Explore relationships with colleagues and how concerns are responded to if raised

Experience of help-seeking

- Explore thoughts/feelings of seeking help
- Explore knowledge of available support and protective factors

What could make things better?

- Explore any realistic changes at individual and organisational level and upstream changes

For peer review only - http://bmjopen.bmj.com/site/about/guidelines.xhtml

REFERENCES

1. NHS Digital, *NHS Workforce Statistics - January 2020*, in *NHS Hospital & Community Health Service (HCHS) monthly workforce statistics*. 2020, Health and Social Care Information Centre.https://digital.nhs.uk/data-and-information/publications/statistical/nhs-workforce-statistics/january-202011/06/2020
2. Brooks, S.K., C. Gerada, and M. Chalder, *Review of literature on the mental health of doctors: Are specialist services needed?* Journal of Mental Health, 2011: p. 1-11.
3. Department of Health, *Mental health and ill health in doctors* 2008, Department of Health: London. http://www.em-online.com/download/medical_article/36516_DH_083090%5B1%5D.pdf 10/03/2021
4. Hawton, K., et al., *Risk factors for suicide in individuals with depression: A systematic review.* Journal of Affective Disorders, 2013. **147**(1): p. 17-28.
5. Menon, N.K., et al., *Association of Physician Burnout With Suicidal Ideation and Medical Errors.* JAMA Network Open, 2020. **3**(12): p. e2028780-e2028780.
6. Dutheil, F., et al., *Suicide among physicians and health-care workers: A systematic review and meta-analysis.* PloS one, 2019. **14**(12): p. e0226361-e0226361.
7. Milner, A., et al., *Suicide among emergency and protective service workers: A retrospective mortality study in Australia, 2001 to 2012.* Work, 2017. **57**(2): p. 281-287.
8. Schernhammer, E.S. and G.A. Colditz, *Suicide rates among physicians: a quantitative and gender assessment (meta-analysis).* American Journal of Psychiatry, 2004. **161**(12): p. 2295-2302.
9. Beyondblue, *The Mental Health of Doctors: A Systematic Literature Review.* 2010.https://www.beyondblue.org.au/docs/default-source/research-project-files/bl1132-report---nmhdmss-full-report_web10/03/2021
10. PHP, *The First Five Years of the NHS Practitioner Health Programme*. 2016. https://www.practitionerhealth.nhs.uk/media/content/files/PHP-report-web%20version%20final(1).pdf.10/03/21
11. Balme, E., *Doctors need to be supported, not trained in resilience.* BMJ: British Medical Journal, 2015. **351**.
12. McKinley, N., et al., *Resilience in medical doctors: a systematic review.* Postgraduate medical journal, 2019. **95**(1121): p. 140-147.
13. Kinman, G. and K. Teoh, *What could make a difference to the mental health of UK doctors? A review of the research evidence*. 2018, Society of Occupational Medicine.https://www.som.org.uk/sites/som.org.uk/files/What_could_make_a_difference_to_the_mental_health_of_UK_doctors_LTF_SOM.pdf9/2/2021
14. Dyrbye, L. and T. Shanafelt, *A narrative review on burnout experienced by medical students and residents.* Med Educ, 2016. **50**(1): p. 132-49.
15. Hayes, B., et al., *What's up doc? A national cross-sectional study of psychological wellbeing of hospital doctors in Ireland.* BMJ Open, 2017. **7**(10).
16. Smith, S.E., et al., *Foundation Year 2 doctors' reasons for leaving UK medicine: an in-depth analysis of decision-making using semistructured interviews.* BMJ Open, 2018. **8**(3).

For peer review only - http://bmjopen.bmj.com/site/about/guidelines.xhtml

17. Hoffman, R.I. and A.D. Bonney, *Junior doctors, burnout and wellbeing Understanding the experience of burnout in general practice registrars and hospital equivalents.* 2018.
18. Casey, D. and K.A. Choong, *Suicide whilst under GMC's fitness to practise investigation: Were those deaths preventable?* Journal of Forensic and Legal Medicine, 2016. **37**: p. 22-27.
19. Laarman, B.S., et al., *How do doctors in the Netherlands perceive the impact of disciplinary procedures and disclosure of disciplinary measures on their professional practice, health and career opportunities? A questionnaire among medical doctors who received a disciplinary measure.* BMJ Open, 2019. **9**(3): p. e023576.
20. Bourne, T., et al., *The impact of complaints procedures on the welfare, health and clinical practise of 7926 doctors in the UK: a cross-sectional survey.* BMJ Open, 2015. **5**(1): p. e006687.
21. Nash, L., et al., *Psychological morbidity in Australian doctors who have and have not experienced a medico-legal matter: cross-sectional survey.* Aust N Z J Psychiatry, 2007. **41**(11): p. 917-25.
22. NHS., *Junior doctor morale. Understanding best practice working environments*. 2017, Health Education England.https://www.hee.nhs.uk/our-work/doctors-training/listening-doctors-training
23. Petrie, K., et al., *Working hours, common mental disorder and suicidal ideation among junior doctors in Australia: a cross-sectional survey.* BMJ open, 2020. **10**(1).
24. West, C.P., et al., *Association of resident fatigue and distress with perceived medical errors.* Jama, 2009. **302**(12): p. 1294-1300.
25. BMA, *BMA Fatigue and Facilities Charter.* 2018. https://www.bma.org.uk/media/1076/bma-fatigue-and-facilities-charter_july2018.pdf 10/03/21
26. Gunasingam, N., et al., *Reducing stress and burnout in junior doctors: the impact of debriefing sessions.* Postgraduate Medical Journal, 2015. **91**(1074): p. 182-187.
27. Flanagan, E., et al., *Reflection for all healthcare staff: a national evaluation of Schwartz rounds.* Journal of Interprofessional Care, 2020. **34**(1): p. 140-142.
28. Bakker, A.B. and E. Demerouti, *Job demands-resources theory: Taking stock and looking forward.* J Occup Health Psychol, 2017. **22**(3): p. 273-285.
29. van der Doef, M. and S. Maes, *The Job Demand-Control (-Support) model and psychological well-being: A review of 20 years of empirical research.* Work & Stress, 1999. **13**(2): p. 87-114.
30. Spiers, J., et al., *Barriers, facilitators, and survival strategies for GPs seeking treatment for distress: a qualitative study.* Br J Gen Pract, 2017. **67**(663): p. e700-e708.
31. Kay, M., et al., *Doctors as patients: a systematic review of doctors' health access and the barriers they experience.* British Journal of General Practice, 2008. **58**(552): p. 501-508.
32. Shanafelt, T.D., et al. *Healing the professional culture of medicine*. in *Mayo Clinic Proceedings*. 94:8 1556-1566 2019.
33. Schein, E.H., & Schein, P. A *The corporate culture survival guide*. 2nd edition. 2009: John Wiley & Sons.
34. Charmaz, K., *Constructionism and the grounded theory method.* Handbook of constructionist research, 2008. **1**: p. 397-412.

For peer review only

35. PHP. *Accessing the Service*. 2020 10/03/2021]; Available from: https://www.practitionerhealth.nhs.uk/accessing-the-service.
36. Saunders, B., et al., *Saturation in qualitative research: exploring its conceptualization and operationalization.* Quality & quantity, 2018. **52**(4): p. 1893-1907.
37. Braun, V. and V. Clarke, *Using thematic analysis in psychology.* Qualitative research in psychology., 2006. **3**(2): p. 77-101.
38. Charmaz, K., *Constructing grounded theory. A practical guide through qualitative analysis*. 2006, London: Sage.
39. Patton, M., *Enhancing the Quality and Credibility of Qualitative Analysis.* HSR, 1999. **34**(5).
40. Tong, A., P. Sainsbury, and J. Craig, *Consolidated criteria for reporting qualitative research (COREQ): a 32-item checklist for interviews and focus groups.* International journal for quality in health care, 2007. **19**(6): p. 349-357.
41. Gottlieb, M., et al., *Impostor syndrome among physicians and physicians in training: A scoping review.* Med Educ, 2020. **54**(2): p. 116-124.
42. Garelick, A.I., *Doctors' health: stigma and the professional discomfort in seeking help.* The Psychiatrist, 2012. **36**(3): p. 81-84.
43. Henderson, M., et al., *Shame! Self-stigmatisation as an obstacle to sick doctors returning to work: a qualitative study.* BMJ Open, 2012. **2**(5).
44. van Pelt, F., *Peer support: healthcare professionals supporting each other after adverse medical events.* Quality and Safety in Health Care, 2008. **17**(4): p. 249.
45. Bruce, S.M., H.M. Conaglen, and J.V. Conaglen, *Burnout in physicians: a case for peer-support.* Intern Med J, 2005. **35**(5): p. 272-8.
46. Shapiro, J. and P. Galowitz, *Peer Support for Clinicians: A Programmatic Approach.* Acad Med, 2016. **91**(9): p. 1200-4.
47. Borrill, C., et al., *Team working and effectiveness in health care.* British Journal of Healthcare Management, 2000. **6**(8): p. 364-371.
48. West, M.A. and L. Markiewicz, *Effective team working in health care.* The Oxford handbook of health care management, 2016: p. 231-254.
49. GOV.UK., *NHS staff management and health service quality.* 2019. https://assets.publishing.service.gov.uk/government/uploads/system/uploads/attachment_data/file/215455/dh_129656.pdf. 03/10/2021
50. *MIND, Introduction to mentally healthy workplaces* 2020.
51. Samsudin, E.Z., et al., *Organisational antecedents of workplace victimisation: The role of organisational climate, culture, leadership, support, and justice in predicting junior doctors' exposure to bullying at work.* The International Journal of Health Planning and Management, 2020. **35**(1): p. 346-367.
52. King's Fund, *NHS leadership and culture: our position.* 2020. https://www.kingsfund.org.uk/projects/positions/NHS-leadership-culture. 10/03/2021
53. West, M. and D. Coia. *Caring for doctors Caring for patients*. 2019.
54. HEE, *Supporting Educators and Learners: Handbook for Educational Supervisors*. 2017.https://www.hee.nhs.uk/sites/default/files/documents/Educational%20Supervisor%20Handbook%20v2.0_draft.pdf09/06/2020
55. Lanius, R.A., E. Vermetten, and C. Pain, *The impact of early life trauma on health and disease: The hidden epidemic*. 2010: Cambridge, UK.

For peer review only - http://bmjopen.bmj.com/site/about/guidelines.xhtml

56. Crumpei, I. and I. Dafinoiu, *The relation of clinical empathy to secondary traumatic stress.* Procedia-Social and Behavioral Sciences, 2012. **33**: p. 438-442.
57. Leigh-Hunt, N., et al., *An overview of systematic reviews on the public health consequences of social isolation and loneliness.* Public Health, 2017. **152**: p. 157-171.
58. Carrieri, D., et al., *Optimising strategies to address mental ill-health in doctors and medical students: 'Care Under Pressure' realist review and implementation guidance.* BMC Medicine, 2020. **18**(1): p. 76.
59. Steffens, N.K., et al., *A Meta-Analytic Review of Social Identification and Health in Organizational Contexts.* Personality and Social Psychology Review, 2016. **21**(4): p. 303-335.
60. Cook, J. and T. Wall, *New work attitude measures of trust, organizational commitment and personal need non-fulfilment.* Journal of Occupational Psychology, 1980. **53**(1): p. 39-52.
61. Leiter, M.P., A. Day, and L. Price, *Attachment styles at work: Measurement, collegial relationships, and burnout.* Burnout Research, 2015. **2**(1): p. 25-35.
62. Seidler, Z.E., et al., *The role of masculinity in men's help-seeking for depression: A systematic review.* Clinical Psychology Review, 2016. **49**: p. 106-118.

For peer review only

For peer review only - http://bmjopen.bmj.com/site/about/guidelines.xhtml

Consolidated criteria for reporting qualitative studies (COREQ): 32-item checklist

Developed from:
Tong A, Sainsbury P, Craig J. Consolidated criteria for reporting qualitative research (COREQ): a 32-item checklist for interviews and focus groups. *International Journal for Quality in Health Care*. 2007. Volume 19, Number 6: pp. 349 – 357

No. Item	Guide questions/description	Reported on Page #
**Domain 1: Research team and reflexivity**		
Personal Characteristics		
1. Interviewer/facilitator	Which author/s conducted the interview or focus group?	Page 8
2. Credentials	What were the researcher's credentials? E.g. PhD, MD	Page 8
3. Occupation	What was their occupation at the time of the study?	Page 8
4. Gender	Was the researcher male or female?	Page 8
5. Experience and training	What experience or training did the researcher have?	Page 8
Relationship with participants		
6. Relationship established	Was a relationship established prior to study commencement?	Page 7 .
7. Participant knowledge of the interviewer	What did the participants know about the researcher? e.g. personal goals, reasons for doing the research	Page 7
8. Interviewer characteristics	What characteristics were reported about the inter viewer/facilitator? e.g. Bias, assumptions, reasons and interests in the research topic	Page 9

For peer review only

Domain 2: study design		
Theoretical framework		
9. Methodological orientation and Theory	What methodological orientation was stated to underpin the study? e.g. grounded theory, discourse analysis, ethnography, phenomenology, content analysis	Page 7
Participant selection		
10. Sampling	How were participants selected? e.g. purposive, convenience, consecutive, snowball	Page 7/8
11. Method of approach	How were participants approached? e.g. face-to-face, telephone, mail, email	Page 7
12. Sample size	How many participants were in the study?	Page 9
13. Non-participation	How many people refused to participate or dropped out? Reasons?	None
Setting		
14. Setting of data collection	Where was the data collected? e.g. home, clinic, workplace	Page 8 .
15. Presence of non-participants	Was anyone else present besides the participants and researchers?	No
16. Description of sample	What are the important characteristics of the sample? e.g. demographic data, date	Page 20/21
Data collection		
17. Interview guide	Were questions, prompts, guides provided by the authors? Was it pilot tested?	Page 22
18. Repeat interviews	Were repeat interviews carried out? If yes, how many?	No, due to lack of resources
19. Audio/visual recording	Did the research use audio or visual recording to collect the data?	Page 8

For peer review only

20. Field notes	Were field notes made during and/or after the interview or focus group?	Yes, Page 8
21. Duration	What was the duration of the inter views or focus group?	Page 9
22. Data saturation	Was data saturation discussed?	Yes, page 9
23. Transcripts returned	Were transcripts returned to participants for comment and/or correction?	No, due to lack of resources
**Domain 3: analysis and findings**		
Data analysis		
24. Number of data coders	How many data coders coded the data?	Page 9
25. Description of the coding tree	Did authors provide a description of the coding tree?	No
26. Derivation of themes	Derived from the data?	Yes, page 9
27. Software	What software, if applicable, was used to manage the data?	Page 9
28. Participant checking	Did participants provide feedback on the findings?	No, due to lack of resources
Reporting		
29. Quotations presented	Were participant quotations presented to illustrate the themes/findings? Was each quotation identified? e.g. participant number	Pages 9-15
30. Data and findings consistent	Was there consistency between the data presented and the findings?	Yes
31. Clarity of major themes	Were major themes clearly presented in the findings?	Yes
32. Clarity of minor themes	Is there a description of diverse cases or discussion of minor themes?	Yes

For peer review only
